# Cellular and Kaposi's sarcoma-associated herpes virus microRNAs in sepsis and surgical trauma

**DOI:** 10.1038/cddis.2014.515

**Published:** 2014-12-04

**Authors:** S Tudor, D E Giza, H Y Lin, L Fabris, K Yoshiaki, L D'Abundo, K M Toale, M Shimizu, M Ferracin, K B Challagundla, M Angelica Cortez, E Fuentes-Mattei, D Tulbure, C Gonzalez, J Henderson, M Row, T W Rice, C Ivan, M Negrini, M Fabbri, J S Morris, S-C J Yeung, C Vasilescu, G A Calin

**Affiliations:** 1 Department of Experimental Therapeutics, The University of Texas MD Anderson Cancer Center, Houston, TX, USA; 2 Department of Surgery, Fundeni Clinical Hospital, Bucharest, Romania; 3 Department of Biostatistics, The University of Texas, MD Anderson Cancer Center, Houston, TX, USA; 4 Department of Pharmacy, The University of Texas, MD Anderson Cancer Center, Houston, TX, USA; 5 Laboratory for Technologies of Advanced Therapies and Department of Morphology, Surgery and Experimental Medicine, University of Ferrara, Ferrara, Italy; 6 Departments of Pediatrics and Molecular Microbiology & Immunology, University of Southern California, Keck School of Medicine, Norris Comprehensive Cancer Center, Children's Center for Cancer and Blood Diseases, Children's Hospital Los Angeles, Los Angeles, CA, USA; 7 Department of Molecular and Cellular Oncology, Molecular Genetics of Cancer Training Program, The University of Texas MD Anderson Cancer Center, Houston, TX, USA; 8 Intensive Care Unit and Department of Anesthesiology, Fundeni Clinical Hospital, Bucharest, Romania; 9 Department of Emergency Medicine, The University of Texas, MD Anderson Cancer Center, Houston, TX, USA; 10 The Center for RNA Interference and Non-coding RNAs, The University of Texas, MD Anderson Cancer Center, Houston, TX, USA; 11 Department of Endocrine Neoplasia and Hormonal Disorders, The University of Texas, MD Anderson Cancer Center, Houston, TX, USA

## Abstract

Once a patient is in septic shock, survival rates drop by 7.6% for every hour of delay in antibiotic therapy. Biomarkers based on the molecular mechanism of sepsis are important for timely diagnosis and triage. Here, we study the potential roles of a panel of cellular and viral miRNAs as sepsis biomarkers. We performed genome-wide microRNA (miRNA) expression profiling in leukocytes from septic patients and nonseptic controls, combined with quantitative RT-PCR in plasmas from two cohorts of septic patients, two cohorts of nonseptic surgical patients and healthy volunteers. Enzyme-linked immunosorbent assay, miRNA transfection and chromatin immunoprecipitation were used to study the effects of Kaposi sarcoma herpes virus (KSHV) miRNAs on interleukin's secretion. Differences related to sepsis etiology were noted for plasma levels of 10 cellular and 2 KSHV miRNAs (miR-K-10b and miR-K-12-12*) between septic and nonseptic patients. All the sepsis groups had high KSHV miRNAs levels compared with controls; Afro-American patients had higher levels of KSHV-miR-K12-12* than non-Afro-American patients. Both KSHV miRNAs were increased on postoperative day 1, but returned to baseline on day 7; they acted as direct agonists of Toll-like receptor 8 (TLR8), which might explain the increased secretion of the IL-6 and IL-10. Cellular and KSHV miRNAs are differentially expressed in sepsis and early postsurgical patients and may be exploited for diagnostic and therapeutic purposes. Increased miR-K-10b and miR-K12-12* are functionally involved in sepsis as agonists of TLR8, forming a positive feedback that may lead to cytokine dysregulation.

Sepsis kills over 10 000 people/day worldwide. Just in the United States, 750 000 sepsis cases occur annually, killing 215 000/year. Sepsis mortality rates range from 20 to 50% in the United States^1^ and from 14 to 41% in Europe.^2^ The reported rate of sepsis in surgical patients is ~5% with an associated mortality rate of 18%.^3^ The physiology of sepsis in surgical patients is believed to differ from medical patients because of the immune modulation associated with surgery and trauma.^3^ Sepsis is clinically diagnosed by the presence of infection and the systemic inflammatory response syndrome (SIRS). The immune system responds, regardless of the cause of sepsis, by secreting inflammatory cytokines (for example, interleukin (IL)-6 and IL-10), dysregulation of which may lead to organ failure and death.^4^ The innate immune system through Toll-like receptors (TLRs) recognize a variety of pathogen-breakdown products such as peptidoglycans, lipopeptides and lipopolysaccharides (LPS) and so on. TLR signal transduction may involve microRNAs (miRNAs), which are short noncoding transcripts that regulate protein-coding gene expression.^5^ Viral single-stranded RNA (ssRNA) binding to TLR 7/TLR8 on dendritic cells and B lymphocytes activate them and increase cytokine production^6^ in a fashion similar to the involvement of circulating miRNAs in cancer metastasis^7^ and in Alzheimer disease.^8^

Early specific intervention in sepsis is crucial for improved outcome; once a patient is in septic shock, survival drops by 7.6% for every hour that antibiotic therapy is delayed.^9^ Many biomarkers have been proposed for sepsis.^10, 11^ Circulating miRNAs seem to be ideal biomarkers owing to their chemical stability in plasma or serum.^12^ MiR-146a, miR-150, miR-223, miR-574-5p and a panel of six plasma miRNAs were found to be potential biomarkers for sepsis.^13^

Apart from cellular miRNAs, DNA virus genomes encode miRNAs to regulate biological functions of the infected host.^14^ Kaposi's sarcoma-associated herpes virus (KSHV, also known as human herpes virus 8) has minimal viral gene expression during latency but has lytic replication upon reactivation, resulting in viral persistence and host-to-host transmission. Several lines of evidence led us to hypothesize that KSHV reactivation may be involved in sepsis: first, some patients co-infected with human immunodeficiency virus and KSHV but without multicentric Castleman's disease develop SIRS;^15^ second, latent KSHV infection increases vascular permeability;^16^ third, KSHV encodes miRNAs that increase IL-6 and IL-10 secretion by leukocytes and macrophages;^17^ and fourth KSHV mainly infects endothelial cells and B lymphocytes, which express multiple TLRs.

In this study, we analyzed specific miRNAs as biomarkers of sepsis in plasma samples from a wide range of clinical settings. We also investigated the potential roles of KSHV-derived miRNAs in the postsurgical and septic states and the mechanism of action by which they increase inflammatory cytokines.

## Results

### Patient characteristics and cytokine production

**Figure 1:**
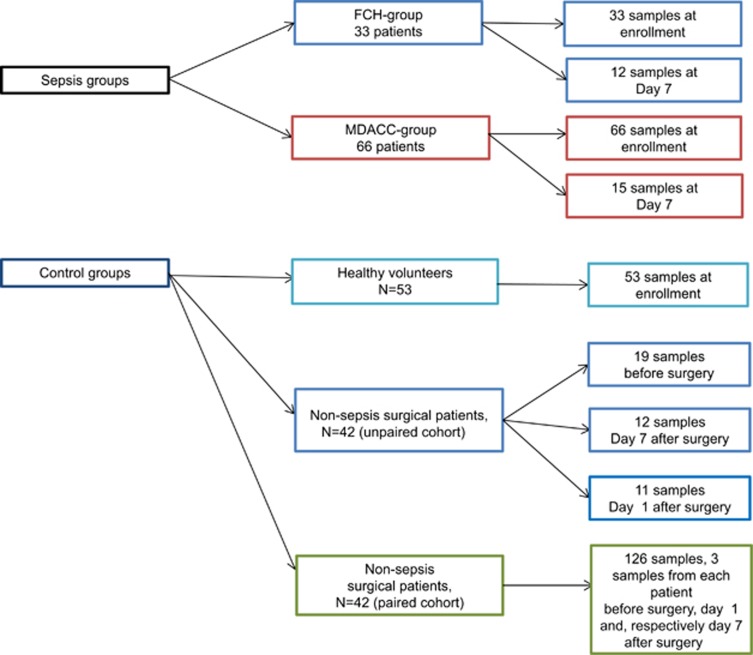
Schematic representation of plasma samples collection from septic, surgical and healthy patients. Work flow representing the plasma sample collection from patients in the three different clinical conditions. For the nonsepsis surgical patients we obtained both plasma and MNC cells

In total, 99 sepsis patients were recruited: 33 from Fundeni Clinical Hospital (FCH) and 66 from The University of Texas MD Anderson Cancer Center (MDA; Table 1). The real-time RT-qPCR for miRNA profiling was normalized using cel-miR-39-3p (ΔCt=Ct^gene^ – Ct^Cel-miR)^, and the ΔCt normalized by cel-miR-39-3p was used in all subsequent analyses because of excellent reproducibility across the different study groups (Supplementary Figure 1). The leading causes of sepsis were major abdominal surgery and pulmonary infection in FCH, neutropenic fever and urinary tract infection in the MDA group. We found significant differences in IL-6 and IL-10 plasma levels between septic patients and healthy individuals (*P*<0.0001 for both) and between the sepsis and septic shock groups (IL-6:*P*=0.0036, IL-10:*P*=0.0096; Supplementary Table 2 – lower table and Supplementary Figure 2). Levels of IL-6 and IL-10 were significantly (*P*<0.05) positively correlated (Spearman's correlation) with SOFA and APACHE-II, and negatively correlated with mean arterial pressure (Supplementary Table 2- upper table). Two cohorts of surgical patients were used, one set of serially repeated samples from 42 individuals (A=before surgery, B=postoperative day 1, C=postoperative day 7) and another set of unrepeated samples (Figure 1).

### miRNA selection by genome-wide profiling

We performed miRNAomic microarray analysis of mononuclear cells (MNCs; primarily leukocytes) from eight septic and eight healthy humans based on the availability of high-quality total RNA as previously described,^18^ and the array data were confirmed by qRT-PCR for several miRNAs.^18^ We further confirmed by qRT-PCR that the two mature forms of miR-342, the 5p and 3p, are both significantly downregulated in the leukocytes from sepsis patients as initially identified by microarray (*P*=0.001 for miR-342-5p and *P*=0.02 for miR-342-3p). The analysis generated 277 human and 17 viral miRNAs that had an expression level above background in at least one sample. On the basis of the criteria that *P*≤0.01 and false discovery rate (FDR) ≤0.1, 1 for differential expression, 10 cellular miRNAs were further investigated by RT-PCR in the clinical study: miR-16, miR-182 and miR-486 were overexpressed and miR-23a, miR-26a, miR-26b, miR-93, miR-146a, miR-150 and miR-342 were downregulated in MNCs from septic patients compared with healthy humans. Two viral miRNAs (KSHV-miR-K12-10b and KSHV-miR-K12-12*) were significantly differentially expressed (Supplementary Table 3 and Supplementary Figure 3) and were also further studied. For the selected miRNAs, efficiency primer amplification was performed, amplifying increasing copy numbers of the selected miRNA (Supplementary Figure 4), to adjust the background at the correct Ct.

### Cellular miRNAs measured by qRT-PCR in plasma samples of septic patients

**Figure 2:**
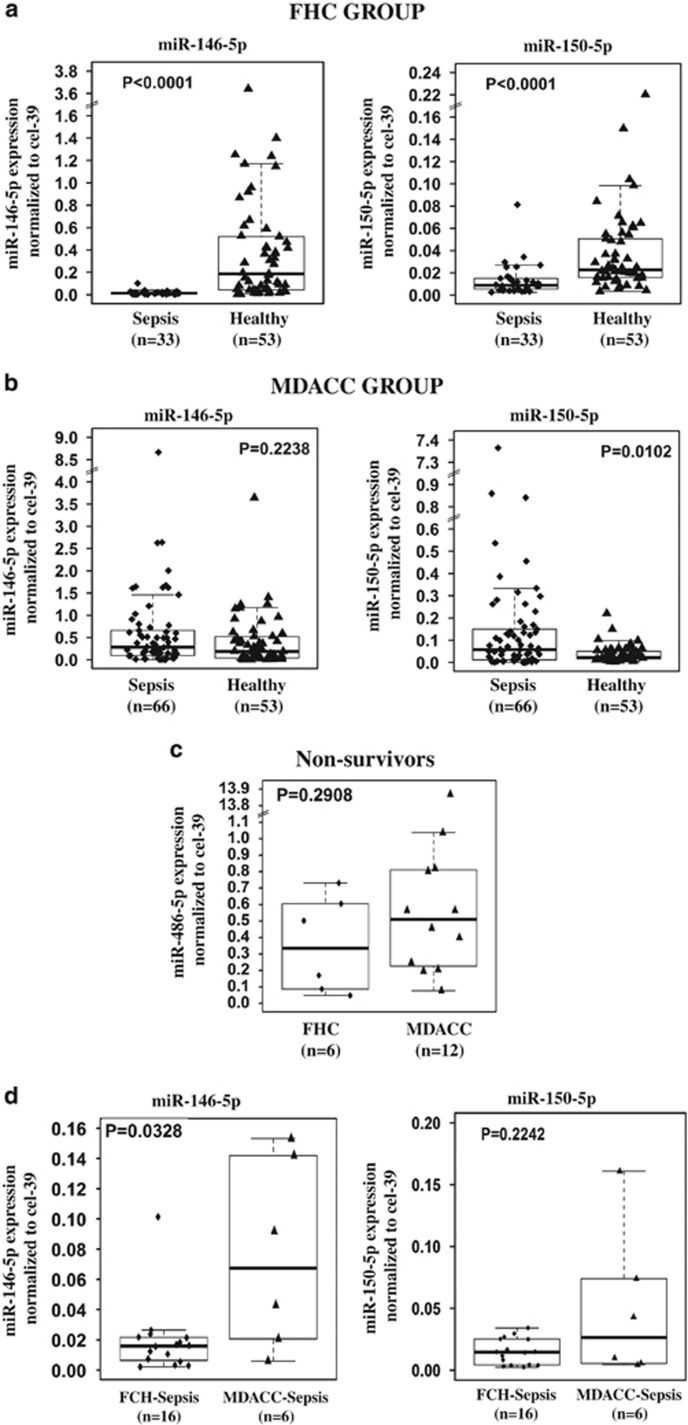
Different cellular miRNAs are significantly deregulated in plasma of sepsis patients compared with normal samples. (**a**,**b**) qRT-PCR showing the comparison of miRNAs between sepsis patients and healthy volunteers in FCH cohort (**a**) and in MDA patients (**b**) at day 1 after septic shock. (**c**) miR-486 expression levels in nonsurgical sepsis patients of the FCH subgroup with pulmonary infection (*n*=9) *versus* expression levels of patients from MDA with pulmonary infection (*n*=12). (**d**) Comparison of miRNAs expression levels between nonsurvivor sepsis patients at FCH and at MDA (***P<0.0001, *P<0.05)

Expression of the 10 cellular miRNAs in FCH sepsis patients was significantly lower than that in healthy subjects (Figure 2a and Supplementary Figure 5). Plasma miR-150 was significantly downregulated in FCH sepsis patients compared with controls on both days 1 and 7 (*P*<0.001 and *P*=0.02, respectively), which confirmed our previous finding in a different cohort.^18^ In MDA sepsis patients, the studied cellular miRNAs were significant higher than that in the healthy controls except for miR-146 and miR-150 (Figure 2b and Supplementary Figure 6). This difference in cellular miRNA expression patterns between the FCH sepsis and MDA sepsis groups might be due to different sepsis etiologies between the groups or due to the technical bias during sample preparation and analysis. To evaluate these possibilities, we compared plasma miRNA levels for different sepsis etiologies within the MDA sepsis group: nonparametric Kruskal–Wallis test showed that patients with neutropenic fever had the lowest levels of miR-23 (*P*=0.045), miR-26a (*P*=0.042), miR-29 (*P*=0.019) and miR-223 (*P*=0.005) and that MDA septic shock patients had the lowest levels of miR-16 (*P*=0.024), miR-182 (*P*=0.043) and miR-486 (*P*=0.009). To exclude the presence of a technical bias, we compared miR-486 levels in pulmonary infections in nonsurgical FCH sepsis patients (*n*=9) with those in MDA sepsis patients with pulmonary infections (*n*=12). There was no significant difference (*P*=0.40) between these subgroups from the two institutions with the same sepsis etiology (Figure 2c). Furthermore, in nonsurvivors, miR-21, miR-23, miR-26a, miR-150, miR-155, miR-182, miR-223, miR-342 and miR-486 had no significant differences (*P*>0.05) between FCH and MDA (Figure 2d and Supplementary Figure 7). The lack of systematic differences across countries for the matched pulmonary infection subgroups provides evidence that the differences in plasma miRNA levels between FCH sepsis and MDA sepsis patients were primarily driven by the differences in sepsis etiologies (Table 1).

### Viral miRNAs measured by qRT-PCR in plasma samples of septic and surgical patients

**Figure 3:**
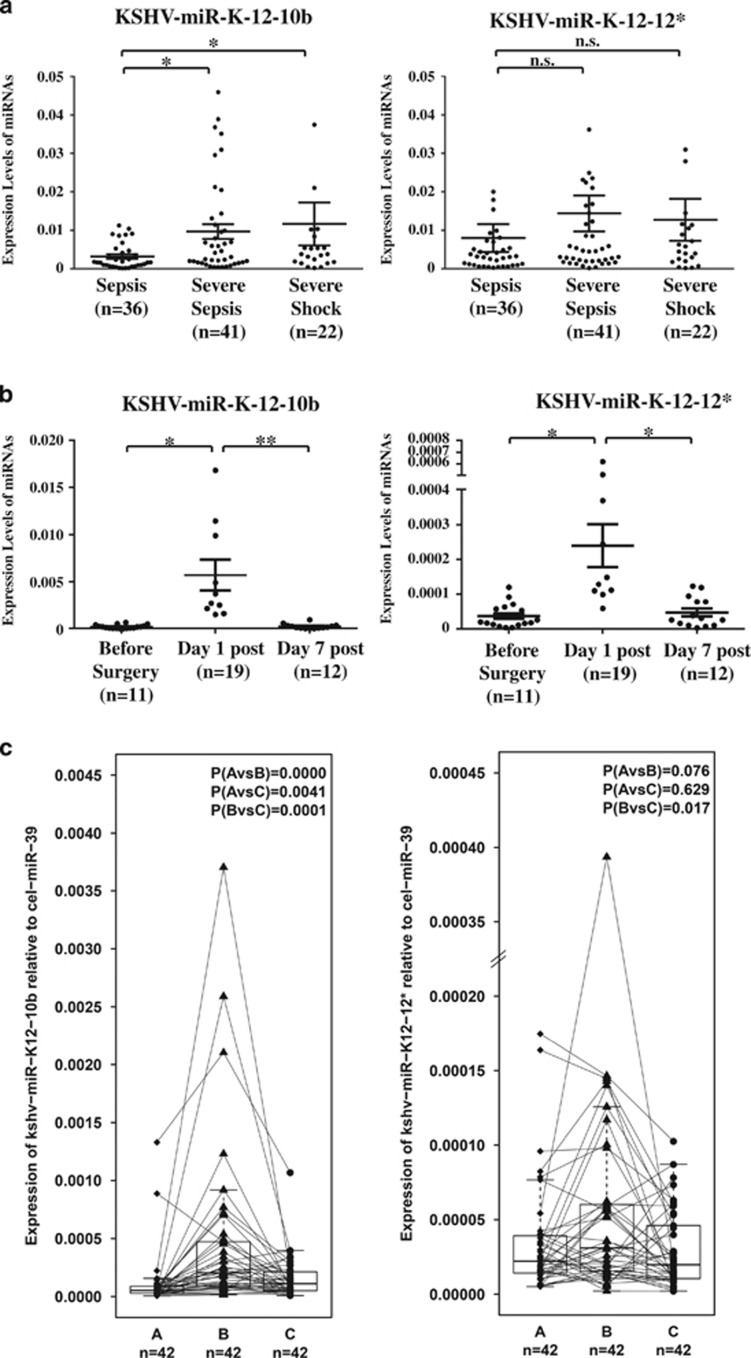
Viral miRNAs are upregulated in plasma of patients after septic shock. (**a**) qRT-PCR showing the expression levels of KSHV-miR-K12-10b and KSHV-miR-K12-12* in the patients, divided for sepsis type (**P<001, *P<05). (**b**) qRT-PCR showing the expression levels of KSHV-miR-K12-10b before surgery and on day 1 and day 7 after surgery in the non-paired set of patients. (**c**) qRT-PCR showing the expression levels of KSHV-miR-K12-10b before surgery and on day 1 and day 7 after surgery in the paired cohort of patients (A=preoperative samples, B=day 1 post surgery samples, C=day 7 post surgery samples)

We found no significant differences between the septic groups studied in either KSHV-miR-K12-10b (*P*=0.178) or KSHV-miR-12-12 *(*P*=0.082; Figure 3a). Also, the proportion of patients with KSHV miRNAs expression (Ct<35) was significantly higher in sepsis patients than healthy controls (Supplementary Table 4). Because surgical trauma can trigger SIRS, we measured plasma levels of KSHV miRNAs preoperatively and on days 1 and 7 after surgery in two nonseptic surgical cohorts: a cohort of 42 patients with unpaired samples and another one with 42 patients with serial samples (clinical data in Supplementary Table 1). Plasma KSHV-miR-K12-10b was very low preoperatively; it increased on postoperative day 1 but returned to preoperative levels by day 7 (Figure 3b (unpaired samples), Figure 3c (serial samples) and Supplementary Figure 8 (cel-miR-39 control for serial samples)). In the serial samples, 11.9% of the surgical patients expressed viral miRNAs preoperatively, whereas on day 1 after surgery, the expression (Ct<35) of the two KSHV miRNAs was detected in 71.42% of patients. In paired MNCs the percentage of viral miRNAs expression was slightly increased (data not shown). We compared the plasma levels of both viral miRNAs in classical open abdominal surgery with minimally invasive (laparoscopic and robotic) surgery. We observed near-significant (0.1>*P*>.05) increases in KSHV miRNAs for the patients who underwent classical surgery, but further investigation is required as the size of this minimally invasive surgical subgroup was small (*n*=8; Supplementary Figure 9). We also analyzed the plasma levels of the two KSHV miRNAs in cancer and noncancer patients with surgical indications (Supplementary Figure 10). Higher expression of KSHV-miR-K12-10b on postoperative day 1 was also found in patients with cancer who underwent classical open abdominal surgery (Supplementary Figure 10 a–c). The expression level of viral miRNAs in surgical patients suggests that surgical trauma triggered KSHV reactivation. When we examined racial differences in the plasma levels of septic patients in the MDA sepsis group, Afro-American patients had significantly higher levels of KSHV-miR-K12-12* (*P***=**0.014) and KSHV-miR-K12-10b (*P*=0.009) than non-Afro-Americans (Supplementary Table 5 and Supplementary Figure 11).

We then analyzed the expression levels of Kaposi sarcoma mRNAs in the serial samples of the nonseptic cohort analyzed in Figure 3c. We aimed to analyze if the patients infected from KSHV dispayed a latent or a lytic infection, and to investigate whether the surgery itself is able to reactivate the viral lytic replication in patients with a latent infection. Real-time PCR analysis of two lytic (NUT-1 and K7) and two latent (LNA and FLIP) KSHV genes display that about half of the patients have detectable expression of these transcripts following the same trend of KSHV-miR-K12-12* and KSHV-miR-K12-10b expression reported in Figure 3c. We found that 50% of patients were positive for the viral NUT-1 lytic transcript (with a Ct mean of 29.7 ±0.5) and a significant positive correlation between KSHV-miR-K-12-10b and NUT-1 expression (Supplementary Figure 12). This further demonstrates that in patients with previous KSHV infection, its lytic reactivation following surgery procedure could significantly influence the outcome of the patients.

### Correlations of miRNA expression with the clinical severity of sepsis by univariate and multivariate analyses

The results of pair-wise analyses of the correlations between the 12 miRNA demographic and clinical characteristics are presented in Supplementary Figure 13. Plasma levels of all 10 cellular miRNAs were negatively correlated with APACHE-II and SOFA (Spearman correlation coefficients (Rho) <−0.21, *P* <0.0001). All the negative correlations remained significant (*P* <0.0006) after Bonferroni adjustment. Univariate analyses also showed that the white blood cell (WBC) count was positively correlated with KSHV-miR-K12-10b and KSHV-miR-K12-12*. These correlations remained significant (*P*<0.0005) after Bonferroni adjustment. Considering each miRNA separately as a predictor of APACHE-II (Supplementary Table 6) using a multivariate model for institution (FCH *versus* MDA), age and sepsis etiology, we found that miR-23, miR-26a, miR-26b, miR-146, miR-150, miR-342 and KSHV-miR-K12-10b were each significantly (*P* <0.05) associated with APACHE-II. However, after Bonferroni adjustment for multiple comparisons, none of the miRNAs remained significant predictors of APACHE-II. Only KSHV-miR-K12-10b remained significant (*P* <0.0001) in a multivariate model including all seven afore mentioned miRNAs. Specifically, APACHE-II decreased by 1.1 per unit of the ΔCt value increase for miRNA expression. Similarly, in the multivariate analysis of SOFA using a model including institution (FCH *versus* MDA), age and sepsis etiologies, we found that miR-23, miR-26a, miR-26b, miR-146, miR-150, miR-342 were significantly (*P* <0.05) associated with SOFA, but none of them remained significant after Bonferroni adjustment. When these six miRNAs were included in the same multivariate model, only KSHV-miR-K-12-10b remained significant (*P*=0.035). Specifically, SOFA decreased by 56% for one unit increase of the ΔCt value for miRNA expression. When sepsis cases were analyzed for associations between miRNAs and death, even though miR-146, miR-16, miR-182, miR-23, miR-26a, miR-26b, miR-93 and miR-342 showed significant (*P* <0.002) association with death within a month of study enrollment in univariate analysis, none of the 12 miRNAs were significant (*P*>0.07) in a multivariate model that included institution and APACHE-II as covariates. Therefore, miR-23, miR-26a, miR-26b, miR-146, miR-150, miR-342 and especially KSHV-miR-K12-10b were predictive of sepsis severity (as reflected by APACHE-II and SOFA), but they were not independent predictors of sepsis mortality in the presence of APACHE-II.

### KSHV miRNAs as direct agonists of TLR8 and inducers of cytokine production

**Figure 4:**
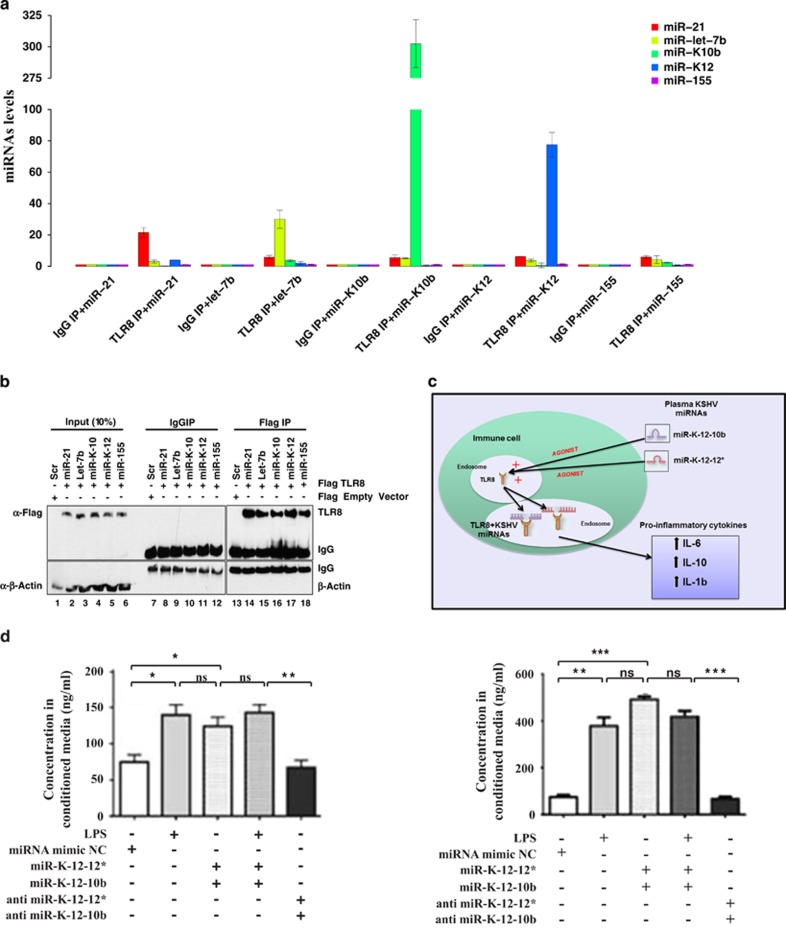
KSHV miRNAs are direct agonists of TLR8 and they increase cytokine production when co-transfected in a histiocytic lymphoma cell line. (**a**) Upper panel: Levels of miRNAs expressed as fold change relative to baseline control determined by quantitative RT-PCR to detect the miR-21 (red), KSHV miR-K12-10b (dark green), KSHV miR-K12-12* (blue). miR-155 (purple) and miR-let-7b (light green) in following samples as grouped from left to right: empty vector transfected HEK293 cells treated with scrambled miRNAs (EV SCR), FLAG-TLR8 HEK293 cells treated with the related miRNA. The experiment was done in duplicates. (**b**) Immunoprecipitation analysis of the experiment shown in **a**. Immunoblotting for actin serves as control for cell content and specificity of immunoprecipitation. The cell samples are as indicated by the plasmid vectors used for transfection and the miRNAs they were incubated with. (**c**) Schema representing plasma viral miRNAs acting as direct agonists on TLR8 receptor and leading to increased secretion of interleukins (IL-6, IL-10 and IL-1b). (**d**) KSHV-miR-K12-10b and KSHV-miR-K12-12* increases the secretion of IL-6 and IL-10. Co-transfection of both viral miRNAs induces secretion of IL-6 and, IL-10 at levels comparable to LPS stimulation

To evaluate whether KSHV miRNAs directly interact with TLR8 we determined the levels of miR-K-12-10b and miR-K12-12* by RT-PCR in HEK-293 cells expressing FLAG-TLR8 and treated with miR-K-12-10b, miR-K-12-12*, two positive controls: miR-21^7^ and and let-7b,^8^ and miR-155, which resulted to be a negative control. We used IgG precipitate/each condition as ‘noTLR8' control and we have assessed the levels of all miRNAs for all conditions (IgG *versus* TLR8 IP co-transfected with miR-21, let-7b, K10, K12*, miR-155). Comparable levels of IgG precipitate in the transfected cells and in the immunoprecipitates were confirmed by immunoblotting (Figure 4a). We identified not only that the two KSHV miRNAs are agonists of TLR8, but also that these miRNAs are more potent (about 5–10 times) agonists with respect to human miRNAs.

We evaluated the functional effect of these two KSHV miRNAs on cytokine production (Figure 4b), as the secretion of IL-6 was already shown to be increased after TLR8 activation by some endogenous miRNAs.^7^ Furthermore, KSHV has been reported to induce the secretion of IL-6 and IL-10.^17^ Therefore, we co-transfected histiocytes derived from U937 cells with KSHV-miR-K12-10b and KSHV-miR-K12-12*. The secretion of IL-6 and IL-10 increased after incubation for 24 or 48 h compared with sham-transfected controls, demonstrating that both miRNAs cooperated to increase the secretion of inflammatory cytokines upon exposure to LPS (Figure 4c). Similar results were obtained for IL-1b and IL-6 in culture media conditioned for 24 h using a different microbead-based immunosorbent assay platform (Supplementary Figures 14 and 15). Finally, to prove that the agonistic effect of viral miRNAs on TLR8 induces interleukin secretion, we repeated the above experiments in a lymphoblast model in BCP1 clonal lymphoma cell line, which is positive for KSHV. We confirmed both the interaction with TLR8 and the induction of IL-6 through TLR8 under the KSHV miR activity (Supplementary Figure 16 ). These data confirm the existence of a new mechanism of action of KSHV involved in the secretion of cytokines.

## Discussion

This study examined the potential roles of a panel of cellular and viral miRNAs as sepsis biomarkers, and we reproducibly and reliably standardized quantitative measurements of plasma miRNAs in patients from different institutions (expressed as ratio to cel-miR-39). Plasma level of miR-150 was significantly downregulated in sepsis, corroborating our previous results with a different patient cohort.^18^ Given the facts that miR-150 in B lymphocytes has been shown to be downregulated following activation by IgM-specific antibodies or LPS^19^ and that miR-146 is involved in innate immunity by regulating the inflammatory response after pathogen recognition by TLRs on monocytes and macrophages,^20^ our results further support their roles as plasma sepsis biomarkers. The two KSHV miRNAs can regulate cytokine secretion in U937 and BCP1 cells, and they are correlated with IL-10 in septic patients. Immunosuppression in sepsis may result in altered innate and adaptive immune responses, exposing the patients to reactivation of endogenous viruses and nosocomial infections.^21^ Deregulation of immunity during sepsis can result in uncontrolled inflammation that may deteriorate into host damage and death. Our discovery of increased expression of KSHV miRNAs in sepsis provides a plausible mechanism of positive feedback that can contribute to mortality. KSHV encodes 10 pre-miRNAs from within the latent gene locus and two others (miR-K12-10 and miR-K12-12*) from within the open reading frame of the K12/Kaposin gene.^22^ Multiple KSHV miRNAs activated transcription and secretion of IL-6 and IL-10 by myelomonocytes and macrophages.^17^ In latency, only a fraction of KSHV genes are expressed to maintain the viral episome. In the lytic phase, most viral genes are expressed and new cells are infected by the virus.^17^ Stimulation of TLR-7 and -8 by ssRNA can activate NFSt (neurofibrillary tangles) and IRF-7 (interferon receptor factor 7) to ‘switch' from latency to the lytic phase.^23^ Tissue damage from sepsis or surgical trauma may release host-derived ssRNA, including viral or cellular miRNAs, to activate this ‘switch'. Here we show that KSHV-miR-K-12-10b and miR-K-12-12* bind to TLR8 and this induces the increased secretion of the IL-6 and IL-10 by the cultured BCP1 clonal lymphoma cell line positive for KSHV. Sepsis following traumatic injury is related to the type of injury, the extent of injury and the immune response of the host. Our study design included patients before and after elective surgery, which allowed us to compare samples before and after the state of immunosuppression triggered by surgery. Our discovery of KSHV miRNAs involvement in sepsis may also shift the paradigm in the investigation of geographic variation and racial difference in sepsis mortality. Sepsis mortality rates vary across the United States^24^ and the world.^25^ It may not be coincidence that Texas is in the ‘Sepsis Belt',^24^ given that the seroprevalence of KSHV in Texas blood donors is substantially higher than that for the general US population. Racial disparities in the sepsis incidence and outcome have been noted in subgroups such as posttransplant patients,^26^ cancer patients,^27^ postoperative patients^28^ and neonates.^29^ Although the racial disparities may be attributed to ‘biological differences' between races, the KSHV prevalence in the Afro-American population is at least a plausible contributing factor to high sepsis mortality.

The fact that specific KSHV miRNAs were expressed in most patients on postoperative day 1 may represent another paradigm-shifting finding, as it implies that in human KSHV infection is much wider than that identified by serological tests. However, this study has limitations that include the relatively small sample size and the heterogeneous nature of septic patients (etiology of sepsis, immunocompromised status, KSHV infection status and so on). Important issues to be addressed in future include the ability of cellular miRNAs to distinguish trauma from early sepsis and the potential therapeutic benefit of agents targeting KSHV in patients with sepsis.

## Materials and Methods

### Clinical samples: sepsis patients and control groups

Sepsis patients and control groups were recruited from two institutions: FCH Bucharest, Romania and MDA, Houston, Texas, USA. At FCH, 33 sepsis patients were recruited in the intensive care unit, and each had blood sampling at enrollment; 12 had follow-up sampling after 7 days (Table 1, Figure 1). At MDA, 66 cancer patients with sepsis were recruited from the Emergency Center. Each patient had blood sampling at enrollment and 15 had follow-up sampling after 7 days while they were still hospitalized. The control groups consisted of one healthy group of 53 volunteer blood donors from MDA (Supplementary Table 1), and two cohorts of surgical patients recruited at FCH: the ‘unpaired' training cohort of 42 surgical patients (19 preoperative samples, 11 in day 1 postoperatory and 12 in day 7 postoperatory) and the ‘paired' validation cohort of 42 surgical patients (each with samples before surgery, and postoperative days 1 and day 7). Clinical records were reviewed and SOFA and APACHE-II scores were calculated (Table 1, Figure 1).

### Genome-wide miRNA expression profiling in mononuclear blood cells

Total RNA samples from mononuclear cells (MNCs) were used for hybridization on a human miRNA microarray as described.^18^ The microarrays consisted of 60-mer DNA probes for 540 human and 63 viral miRNAs. Data were analyzed using GeneSpring GX software (v7**·**3; Agilent Technologies, Santa Clara, CA, USA).

### Measurement of cytokines

Mimetics of KSHV-miR-K12-12-5p (also known as KSHV-miR-K12-12*, the name used in our publication) and KSHV-miR-K12-10b and their respective miRNA inhibitors (MirVanaTM mRNA inhibitors, Life Technologies, Grand Island, NY, USA) were transfected with Lipofectamine in human histiocytic lymphoma cell line U-397 and in the BCP1 clonal lymphoma cell line (American Type Culture Collection, Manassas, VA, USA). The plasma levels of human IL-10 and IL-6 were measured by enzyme-linked immunosorbent assay (ELISA) using commercial kits (BD Biosciences, San Jose, CA, USA) and a microbead-based immunosorbent assay platform for measuring 12 cytokines (IL-1b, -2, -4, -5, -6, -7, -8, -10, -12(p70), -13, IFN-γ and TNF-α Millipore, Billerica, MA, USA).

### Statistical analysis

*χ*^2^-test or Fisher's exact test for categorical variables and Wilcoxon rank-sum, or Kruskal–Wallis test for continuous variables, was used to test the differences between groups, where appropriate. Spearman correlation coefficients were used to assess the correlations between continuous variables. Linear regression models were used to evaluate the associations between patient characteristics and miRNA expression with APACHE-II or SOFA in the multivariate setting. Logistic regression analysis was used to assess the multivariate relationship between patient demographics, clinical characteristics and miRNA levels and the probability of death. Bonferroni corrections were used to adjust for multiple testing. SAS version 9. 2 and SPlus version 8. 04 were used to compute all the analyses. To examine the association between response to surgery and miRNA levels, we performed a pair-wise comparison between preoperative phase, postoperative day 1 and day 7. The data were first tested for normality (Shapiro–Wilk normality test), and we concluded that the data were not normal. The nonparametric Wilcoxon-test was thus applied and the Benjamini–Hochberg FDR method was used to correct for the multiple testing results. The analysis was performed in R version 2. 14. 2, http://www.R-project.org/.

Additional details on RNA extraction from plasma samples, reverse transcription (RT) and real-time RT-qPCR profiling, on genome-wide miRNA expression profiling in leukocytes, on ELISA, cytokine release experiments in U-397 and BCP1 cells after KSHV miRNAs transfection, immunoprecipitation of protein-associated RNAs for TLR8 in HEK-293 cells and viral miRNAs measurement by qRT-PCR from TLR8-FLAG-HEK-293 and BCP1 cells is provided in the Supplementary Information.

## Figures and Tables

**Table 1:**
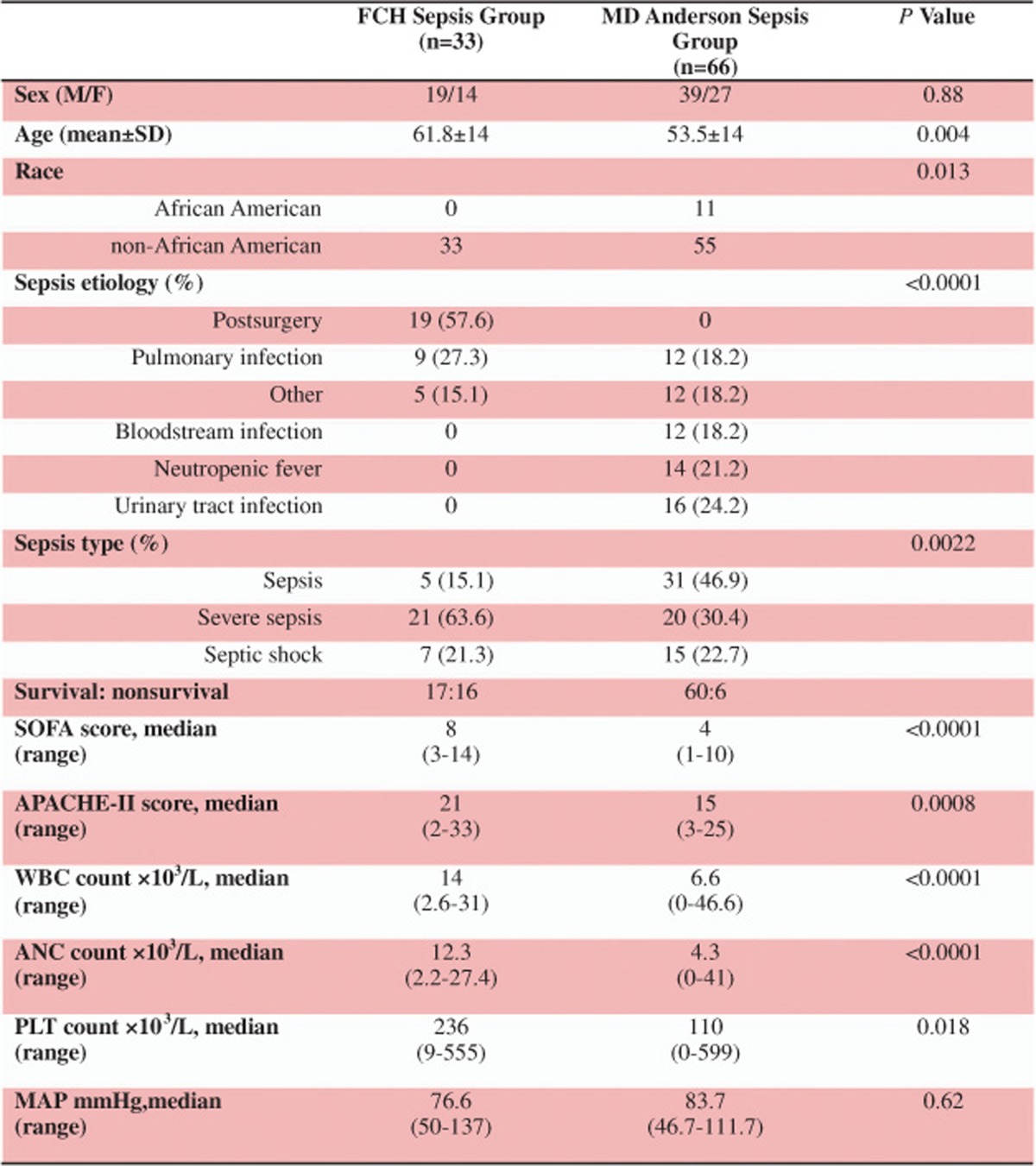
Demographic and clinical data of sepsis patients.

## Abbreviations

FCH: Fundeni Clinical Institute
MDACC: MD Anderson Cancer Center
MDA: MD Anderson Cancer Center
KSHV-miR-12-12*: KSHV-miR-12-12-5p
