# Supplementary material for: Cellular and Kaposi's sarcoma-associated herpes virus microRNAs in sepsis and surgical trauma
Source: Cell Death Dis. 2014 Dec 4;5(12):e1559–. doi: 10.1038/cddis.2014.515 (PMC4649832; doi:10.1038/cddis.2014.515)
Supplement: Supplementary Information [file cddis2014515x1.doc]

Cellular and Kaposi’s Sarcoma-associated Herpesvirus microRNAs

in Sepsis and Surgical Trauma

*Stefan Tudor^a, b^, MD, PhD; Dana Elena Giza^a, b^, MD; Heather Y. Lin^c^ PhD, Linda Fabris ^1^ PhD;*

*Kita Yoshiaki^a^, MD;Lucilla D’Abundo^a^, PhD; Katy M Toale^d^, PharmaD;*

*Masayoshi Shimizu^a^, BS;Manuela Ferracin^e^, PhD; Kishore B. Challagundla^f^, PhD;*

*Maria Angelica Cortez^a^, PhD;Enrique Fuentes-Mattei^g^, PhD; Dan Tulbure^h^, MD;*

*Carmen Gonzalez^i^, MD;Jerry Henderson^i^, MD; Margaret Row^i^, MD; Terry W. Rice^i^, MD; Cristina Ivan^j^, PhD;Massimo Negrini^e^, MD; Muller Fabbri^f^, MD, PhD; Jeffrey S. Morris^c^, PhD;*

*Sai-Ching Jim Yeung^i, k, #^, MD, PhD; Catalin Vasilescu ^b, #^, MD, PhD;*

*George A. Calin^a, j, #^ MD, PhD*

**Supplementary Materials and Methods:**

**Genome-wide miRNA expression profiling in leukocytes**

Sixteen total RNA samples, extracted from leukocytes purified from 8 sepsis patients and 8 “healthy” controls without sepsis (see definition below), were used for hybridization on a human miRNA microarray (G4470A, Agilent Technologies, Austin, TX) as previously described (1).Tot al RNA from leukocytes was isolated using TRIZOL reagent (Invitrogen, Carlsbad, CA) and RNA quality was assessed by Agilent 2100 Bioanalyzer (Agilent Technologies)(1). The microarrays consisted of 60-mer DNA probes for 540 human and for 63 viral miRNAs including Kaposi Sarcoma-associated Herpes Virus (KSHV), Epstein-Barr virus (EBV), and Human Cytomegalic Virus (HCMV) sourced from the Sanger miRBase public database (Release 9.1). One-color miRNA microarray analysis was performed according to the manufacturer’s procedure, and the data were analyzed using GeneSpring GX software, version 12 (Agilent Technologies). Statistical analysis of viral miRNAs was performed using moderated t-test. Cluster analysis was performed using Pearson centered correlation.

**Clinical Samples: Sepsis Patients and Control Groups**

The study subjects were recruited from two institutions: Fundeni Clinical Hospital (FCH), Bucharest, Romania, and The University of Texas MD Anderson Cancer Center (MDA), Houston, Texas. All clinical data and blood samples were obtained from participants who had given written informed consent, according to protocols approved by the FCH Ethics Committee and MD Anderson Institution Review Board. Sepsis was defined as the presence of SIRS and evidence of infection.

At FCH, 33 sepsis patients admitted to the ICU participated. Sepsis was diagnosed according to American College of Chest Physicians and the Society of Critical Care Medicine; each patient had a blood sample drawn at enrollment, and 12 patients had follow-up blood sampling after 7 day (**Table 1 and Figure 1**).The non-sepsis control groups recruited at FCH included a group of 19 non-sepsis surgical patients with 19 samples taken before surgery and 12 samples taken on postoperative day 7, a group of 11 non-sepsis surgical patients with blood samples taken on day 1 after surgery, and a group that included 42 patients who underwent surgery for different pathologies, with samples before surgery and on days 1 and 7 after surgery (**Supplementary Table 1**). The normal healthy control group consisted of 53 healthy volunteer blood donors at MDA (**Supplementary Table 1**);the subjects were previously described (1);they were blood donors at MDA, and “healthy” was defined by the absence of any infection or known medical condition at the time of blood collection.

At MDA, 66 cancer patients with sepsis (SIRS with evidence of infection) ranging from very mild sepsis to septic shock were recruited from the Emergency Center. Each patient had a blood sample drawn at enrollment, and 15 patients had follow-up blood samples collected 7 days after enrollment while they were still hospitalized. All blood samples were collected in 10 mL EDTA tubes, and plasma samples were processed, frozen in aliquots, and stored at −80°C within 24 hours after collection.

In compliance with HIPAA regulations, clinical data were reviewed to obtain information to determine the SOFA and APACHE-II scores and sepsis severity at enrollment (**Table 1**). Sepsis severity was stratified as mild sepsis, severe sepsis, and septic shock. Severe sepsis occurs when there is dysfunction of≥1 organ or system,where septic shock is associated with refractory arterial hypotension despite aggressive fluid resuscitation and with multi-organ dysfunction.

**RNA Extraction from Plasma Samples and Reverse Transcription (RT)**

Total RNAs were extracted from 200 µl of plasma using a total RNA purification kit (NorgenBiotek, Thorold, ON, Canada) and eluted with 50 µl of elution solution. For the normalization of sample-to-sample variation in the RNA isolation step, the *Caenorhabditis elegans* cel-miR-39-3p, (mirVana® miRNA mimic, Applied Biosystems, Foster City, CA); 25 fmol of each in a total volume of 5 µL were added to each denatured sample after mixing the plasma sample with lysis buffer. Total RNA concentrations were measured using a NanoDrop ND-1000 spectrophotometer.

For gene expression analysis, RNA was retro-transcribed with SS III Reverse transcriptase to obtain cDNAs, according to provider’s instruction (Life Technologies). Relative quantification of targets was evaluated by qRT-PCR using SYBR Green dye-containing reaction buffer (SsoAdvanced SYBR SuperMix, Biorad), and applying the formula 2^-(ΔCt)^. Primer efficiency was caluculated before using the primers. The incorporation of the SYBR Green dye into the PCR products was monitored in real time using the CFX384 Real-time PCR Detection System (Biorad). Normalized expression was evaluated by using U6 as housekeeping gene.­­

Primers used were:

LNA FW: GTGACCTTGGCGATGACCTA

LNA RW: CAGGAGATGGAGAATGAGTA

FLIP FW: ATGGCCACTTACGAGGTTCT

FLIP RW: ACGTGGAGAACAGTGAGCTG

K7 FW: ATGGGAACACTGGAGATAAA

K7 RW: TTGGGCAAATCGCAGCTTT

NUT1 FW: GAGCTCTAGGCACGTTAAATTGTC

NUT 1 RW: TAGGCGACAAAGTGAGGTGGC

RNA was reverse transcribed using the TaqMan® miRNA Reverse Kit (Applied Biosystems) in 10μL RT reaction containing 10 ng of RNA, 0.1μL of 100 mM dNTPs , 0.67μL of Multiscribe reverse transcriptase, 1μL of 10× RT buffer, 0.13μL of RNase inhibitor, and 1μL of 5×miRNA-specific stem-loop RT primer (Applied Biosystems).

Reverse transcription was performed in a BioRad DNA engine with the following program: 16°C for 30 min, 42°C for 30 min, 85°C for 5 min, and then 4°C on hold. The cDNA was diluted and stored at −20°C until analysis.

**Construction of vectors and calculation of copy number**

To generate a standard curve for primer validation, target microRNA fragments were amplified and purified using DNA cleanup and concentration kit kit (zymoresearch). Fragments were ligated into plasmid pCR2.1 (Invitrogen), and transformed into Escherichia (E.) coli Top10 competent cells and incubated overnight at 37°C. Recombinants were screened by resistance to ampicillin. Absorbance of the pufified plasmids DNA were measured by a NanoDrop ND-1000 spectrophotometer and the copy number was calculated using the following formula: [X μg/μl plasmid DNA/(plasmid and target DNA length) ×660] × 6.022 × 10^23^ = Y molecular number/μl. X represents the concentration of plasmid DNA and Y represents copy number.

**Real-Time RT-qPCR Profiling and Normalization**

The diluted cDNA (2.5 µL) was used as template in a quantitative PCR (qPCR) reaction with a total final volume of 5 µL. DNA amplification was performed using TaqMan primers/probes specific for each miRNA together with SsoFast™ Probes Supermix (Bio-Rad Laboratories, Hercules, CA). The reaction started with incubation for 3 min at 95°C followed by 40 cycles of 5 sec at 95°C and 30 sec at 60°C. All experiments were performed in duplicate. Ct values beyond the upper limit of the measuring system are imputed as 40. The raw Ct values were normalized by cel-miR-39-3p(ΔCt = Ct^gene^ – Ct^Cel-miR)^ , and the ΔCt normalized by cel-miR-39-3p was used in all subsequent analyses because of excellent reproducibility across different study groups.

**Enzyme-Linked Immunosorbent Assay (ELISA)**

The plasma levels of human IL-10 and IL-6 were measured by ELISA using Human BD OptEIA™ ELISA Kit II for IL-10 and IL-6 (BD Biosciences, Erembodegem, Belgium) according to the manufacturer. Optical density at 450 nm was measured using a Spectra Max Plus plate reader (Molecular Devices, Sunnyvale, CA). The minimum detectable concentrations were 2 pg/mL for IL-10 and 2.2 pg/mL for IL-6. A microbead-based immunosorbent assay platform (Millipore, Inc.), measuring 12 cytokines (IL-1b, -2, -4, -5, -6, -7, -8, -10, -12(p70), -13, IFN-γ, and TNF alpha) was used as a second method for IL measurement.

**Cells and Transfection Conditions for Immunoprecipitation of TLR8 protein-associated RNAs in HEK-293 cells**

***Cell culture***

Human embryonic kidney epithelial 293 cells were cultured in Dulbecco’s modified Eagle’s medium (DMEM) supplemented with 10% fetal bovine serum (FBS), 50 U of penicillin/ml, and 0,1 mg of streptomycin/ml at 37°C in a 5% CO2 humidified atmosphere.

***Transfection***

HEK 293 cells were transfected with Flag empty vector or Flag-TLR8 plasmids (kind gift provided by Dr.Misako Matsumoto, Hokkaido University Graduate School of Medicine, Japan) for 24 hrs using X-treme GENE HP DNA transfection reagent followed by the manufacturer’s protocol (Roche Applied Science) with minor modifications(2). Transfection of miRs carried out using Dotapreagent (Roche Applied Science) for additional 36 hrs according to the manufacturer's protocol. Cells were harvested and separated the pellets into two parts. A major portion of the pellet was used for RNA IP, and the remainder for Western blot assays.

***Immunoprecipitation of protein-associated RNAs (RNA IP)***

RNA-protein complexes immunoprecipitation was performed as described with minor modifications (2). Briefly, cells were lysed in polysomelysis buffer (PLB) (100 mMKCl, 5 mM MgCl2, 10 mM HEPES [pH 7.0], 0.5% Nonidet P-40, 1 mM DTT, 100 U of RNase inhibitor/ml) supplemented with 20 mM EDTA and protease inhibitors on ice for 20 min followed by centrifugation. The supernatants were then diluted (1:10 [vol/vol]) in freshly made NT2 buffer (50 mM Tris [pH 7.4], 150 mM NaCl, 1 mM MgCl2, 0.05% Nonidet P-40, 1 mM DTT, 100 U of RNase inhibitor/ml) supplemented with 20 mM EDTA and protease inhibitors and incubated with α-Flag beads (Sigma) at 4°C for 6 h. The beads were washed five times with NT2 buffer supplemented with protease inhibitors. The bead-bound protein-RNA complexes were then treated with DNase I and proteinase K and eluted twice with NT2 buffer containing 0.1% SDS. RNAs were extracted from the elution with phenol-chloroform and ethanol precipitation followed by reverse transcription and qRT-PCR assay for miRNAs.

***Immunoblot (IB) and immunoprecipitation (IP)***

Cells were lysed in lysis buffer consisting of 50 mMTris-HCl (pH 8.0), 0.5% Nonidet P-40, 1 mM EDTA, 150 mM NaCl, 1 mM phenylmethylsulfonyl fluoride (PMSF), 1mM DTT, 1 μg/ml pepstatin A, and 1mM leupeptin. Equal amounts of clear cell lysate were used for immunoprecipitation analysis. Cell lysates were incubated with α-Flag beads (Sigma) at 4°C for 6 h. Beads were washed with lysis buffer for four times. Bound proteins were detected by IB using α-Flag (Sigma) or α-Actin (Cell Signal Technology) antibodies.

***RNA extraction from RNA-protein complexes for Reverse Transcription (RT)***

RNAs were extracted from the Flag-TLR8 bound proteins as mentioned above in RNA_IP using phenol-chloroform and ethanol precipitation and further reverse transcribed (kit details) and the cDNA was diluted and stored at −20°C.

**Viral miRNAs Measured by qRT-PCR in TLR8-FLAG-HEK-293 cells**

The diluted cDNA (2.5 µL) was used as template in a quantitative PCR (qPCR) reaction with a total final volume of 5 µL. DNA amplification was performed using TaqMan primers/probes specific for each miRNA together with SsoFast™ Probes Supermix )(Bio-Rad Laboratories, Hercules, CA). The reaction started with incubation for 3 min at 95°C followed by 40 cycles of 5 sec at 95°C and 30 sec at 60°C . All experiments were performed in duplicate**.** The levels of miR -21, KSHV-miR-K-12-10b, KSHV-miR-12-12* were measured in HEK 293 cells expressing FLAG-TLR8 and treated with miR-21, KSHV-miR-K-12-10b, KSHV-miR-12-12* by quantitative real time PCR and compared with cells treated with scrambled control. The experiment was done in duplicates; empty vector (EVSCR) was used as a control.

**BCP1 cells transfection with TLR8 siRNA, TLR8 mRNA levels quantification by qRT-PCR and IL-6 detection by ELISA**

BCP1 cells (ATCC® CRL­2294™) were cultured with base medium for ATCC­formulated RPMI­1640 Medium, Catalog No. 30­2001 for 12 hours and then transfected with TLR8 siRNA (smart pool from Dharmacon) for 24 h using silentFect lipid reagent (Bio-Rad) following the manufacturer's protocol. MiR-21 and viral miRNAs (miR-K-12-10b and miR-K-12-12*) were transfected using Dotap reagent (Roche Applied Science) for additional 36 hrs according to the manufacturer's protocol. Cells were then harvested for RNA isolation and the supernatant was saved for IL-6 detection. The RNA extraction, TLR8 mRNA levels quantification by qRT-PCR and the IL-6 release detection are similar to those described for U937 cells.

**Supplementary Tables:**

**Supplementary Table 1.Clinical data for the FCH non-sepsis and healthy control groups.**

|  | **FCH Non-sepsis Control Group**  **(n=42)** | **Healthy Control Group**  **(n=53)** |
| --- | --- | --- |
| Sex, M/F | 22/20 | 25/28 |
| Age (mean±SD) | 57.23±15.05 | 42.41±11.7 |
| Diagnosis |  |  |
| Malignant | 26 |  |
| Non-malignant | 16 |  |
| Surgical approach |  |  |
| Open surgery | 34 |  |
| Minimally invasive surgery | 8 |  |

**Supplementary Table 2. Spearman r correlation between cytokine levels and clinical data of sepsis severity (^**^*P*<.01, ^*^*P*<.05) (upper table) and differences between the 2 sets of sepsis patients (Lower Table).**

|  | **SOFA** | **APACHE II** | **MAP** |
| --- | --- | --- | --- |
| IL-10 | 0.2947^**^ | 0.2925^**^ | -0.3162^**^ |
| IL-6 | 0.2105^*^ | 0.2223^*^ | -0.3681^**^ |

Where MAP = 60-110 mmHg.

|  | **FCH Sepsis Group**  **(n = 33)** | **MDACC Sepsis Group**  **(n = 66)** | ***P* value** |
| --- | --- | --- | --- |
| IL-10, pg/mL, median (range) | 66.3  (10.31-271.6) | 26.9  (3.58-242.8) | .024 |
| IL-6, pg/mL, median (range) | 192  (27-1353) | 114.5  (3.57-1354) | .27 |

**Supplementary Table 3. Microarray data of miRNAs selected for further qRT-PCR analyses in plasma samples.**

|  | **miRBase Name** | **Sepsis versus Controls** | **Fold Change**  **(Sepsis/Control)** | **Parametric**  ***P*-value** | **FDR** |
| --- | --- | --- | --- | --- | --- |
| 1 | hsa-miR-93-5p | Up | 1.49 | .0003 | 0.0163 |
| 2 | hsa-miR-26a-5p | Down | 0.53 | .0004 | 0.0163 |
| 3 | hsa-miR-486-5p | Up | 6.51 | .0010 | 0.0247 |
| 4 | hsa-miR-342-3p | Down | 0.46 | .0019 | 0.0336 |
| 5 | hsa-miR-23a-3p | Down | 0.60 | .0023 | 0.0341 |
| 6 | hsa-miR-16-5p | Up | 1.48 | .0069 | 0.0836 |
| 7 | hsa-miR-150-5p | Down | 0.44 | .0079 | 0.0836 |
| 8 | hsa-miR-146a-5p | Down | 1.83 | .0086 | 0.0836 |
| 9 | hsa-miR-26b-5p | Down | 0.60 | .0098 | 0.08382 |
| 10 | hsa-miR-182-5p | Up | 5.73 | .0105 | 0.0838 |
| 11 | KSHV-miR-K12-10b | Down | 0.03 | .00006 | <0.08 |
| 12 | KSHV-miR-K12-12* | Up | 9.58 | .048 | <0.08 |

**Supplementary Table 4.Comparison of KSHV miRNAs expression between septic and post-surgical patients and healthy controls using Fischer’s test.**

| **Patient**  **Sample** | **KSHV-miR-k12-10b Positive (Ct<35)** | **KSHV-miR-k12-10b Negative(Ct>35)** |  |
| --- | --- | --- | --- |
| Septic patients (MDACC+FCH day 1) | 89(90%) | 10(10%) |  |
| Healthy controls | 0(0%) | 53(100%) | **P<2.2e-16.** |

| **Patient**  **Sample** | **KSHV-miR-k12-12* Positive (Ct<35)** | **KSHV-miR-k12-12* Negative**  **(Ct>35)** |  |
| --- | --- | --- | --- |
| Septic patients (MDACC+FCH day 1) | 32(32%) | 67(68%) |  |
| Healthy controls | 0(0%) | 53(100%) | **P = 1.5e-07** |

**Supplementary Table 5. Comparison of miRNAs measured on day 1 by race for MDA-sepsis patients.**

| **Marker** | **Race** | **n** | **Mean** | **Std** | **Stderr** | **Min** | **Max** | **Median** | ***P*-Value** |
| --- | --- | --- | --- | --- | --- | --- | --- | --- | --- |
| KSHV-miR-K12-12* | Afro-American | 11 | 10.02 | 2.25 | 0.68 | 6.26 | 13.51 | 10.43 | **.014** |
|  | Non-afro-American | 55 | 11.93 | 1.78 | 0.24 | 8.49 | 15.86 | 11.68 | . |
| KSHV-miR-k12-10b | Afro-American | 11 | 6.87 | 2.22 | 0.67 | 3.02 | 10.77 | 6.79 | **.009** |
|  | Non-afro-American | 55 | 8.87 | 1.95 | 0.26 | 4.47 | 12.49 | 9.08 | . |
| miR-16 | Afro-American | 11 | -4.25 | 1.86 | 0.56 | -8.12 | -0.79 | -4.28 | **.023** |
|  | Non-afro-American | 55 | -2.82 | 1.74 | 0.24 | -6.63 | 0.78 | -3.27 | . |
| miR-182 | Afro-American | 11 | 7.60 | 2.93 | 0.88 | 4.57 | 15.16 | 7.28 | .**041** |
|  | Non-afro-American | 55 | 8.90 | 2.42 | 0.33 | 4.46 | 15.65 | 8.74 | . |
| miR-29a | Afro-American | 11 | 2.79 | 2.25 | 0.68 | 0.83 | 8.48 | 2.52 | .**045** |
|  | Non-afro-American | 55 | 4.13 | 2.53 | 0.34 | -0.27 | 11.36 | 3.64 | . |
| miR-486 | Afro-American | 11 | -0.15 | 1.57 | 0.47 | -3.79 | 2.26 | 0.29 | **.014** |
|  | Non-afro-American | 55 | 1.36 | 1.85 | 0.25 | -2.68 | 7.74 | 1.03 | . |
| miR-93 | Afro-American | 11 | -0.03 | 1.68 | 0.51 | -2.73 | 3.89 | -0.14 | **.025** |
|  | Non-afro-American | 55 | 1.56 | 2.72 | 0.37 | -3.03 | 14.52 | 1.04 | . |
| miR-21 | Afro-American | 11 | 0.73 | 2.15 | 0.65 | -1.73 | 6.18 | 0.37 | **.024** |
|  | Non-afro-American | 55 | 2.04 | 2.08 | 0.28 | -2.47 | 6.86 | 1.71 | . |
| miR-26b | Afro-American | 11 | -0.19 | 2.47 | 0.74 | -2.31 | 6.51 | -0.74 | **.034** |
|  | Non-afro-American | 55 | 1.26 | 2.94 | 0.40 | -4.26 | 13.33 | 0.96 | . |

**Supplementary Table 6. Effect of miRNA on APACHE-II in multivariate linear regression model of APACHE-II adjusting for institution, age, and sepsis etiology.***Change of APACHE-II for 1 unit increase of the ∆Ct value (level of a miRNA = -log_2_(2^∆Ct)).The LowerWaldCL and UpperWaldCL are the lower and upper limits of the confidence intervals for the estimates.

| **Parameter** | **Estimate*** | **StdErr** | | **LowerWaldCL** | **UpperWaldCL** | **ChiSq** |
| --- | --- | --- | --- | --- | --- | --- |
| mir_146 | 0.4986 | 0.2220 | 0.0635 | 0.9337 | 5.04 | **0.0247** |
| miR_150 | 0.4711 | 0.2153 | 0.0491 | 0.8931 | 4.79 | **0.0287** |
| miR_486 | 0.3933 | 0.2980 | -0.1907 | 0.9773 | 1.74 | 0.1868 |
| miR_16 | 0.5486 | 0.2895 | -0.0189 | 1.1160 | 3.59 | 0.0581 |
| miR_182 | 0.2640 | 0.2354 | -0.1974 | 0.7253 | 1.26 | 0.2621 |
| miR_23 | 0.3637 | 0.1809 | 0.0091 | 0.7183 | 4.04 | **0.0444** |
| miR_26a | 0.4177 | 0.1840 | 0.0571 | 0.7782 | 5.16 | **0.0232** |
| mir_26b | 0.4571 | 0.2136 | 0.0384 | 0.8759 | 4.58 | **0.0324** |
| miR_93 | 0.3709 | 0.2289 | -0.0776 | 0.8195 | 2.63 | 0.1051 |
| miR_342 | 0.4570 | 0.1971 | 0.0708 | 0.8433 | 5.38 | **0.0204** |
| KSHV_miR_K12_10b | -0.6635 | 0.2583 | -1.1697 | -0.1573 | 6.60 | **0.0102** |
| KSHV_miR_K12_12* | -0.4521 | 0.2612 | -0.9640 | 0.0598 | 3.00 | **0.0834** |

**Supplemental Figures:**


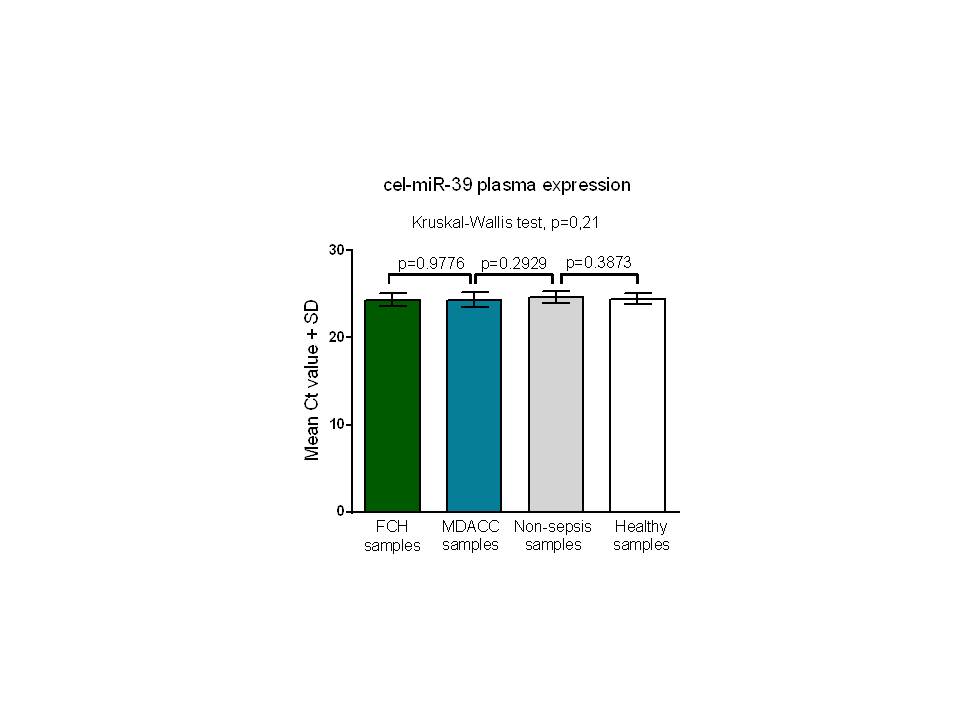


**Supplementary Figure 1. Ct Values of cel-miR-39 are similar across studied groups.**


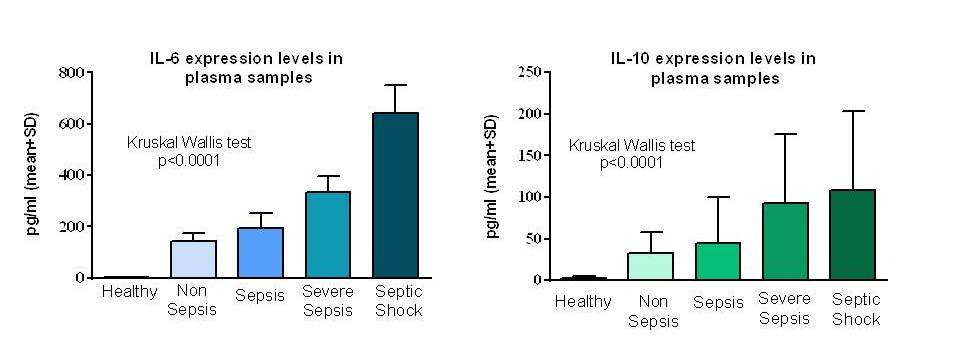


**Supplementary Figure 2. IL-10 and IL-6 plasma levels between sepsis patients and control groups.**


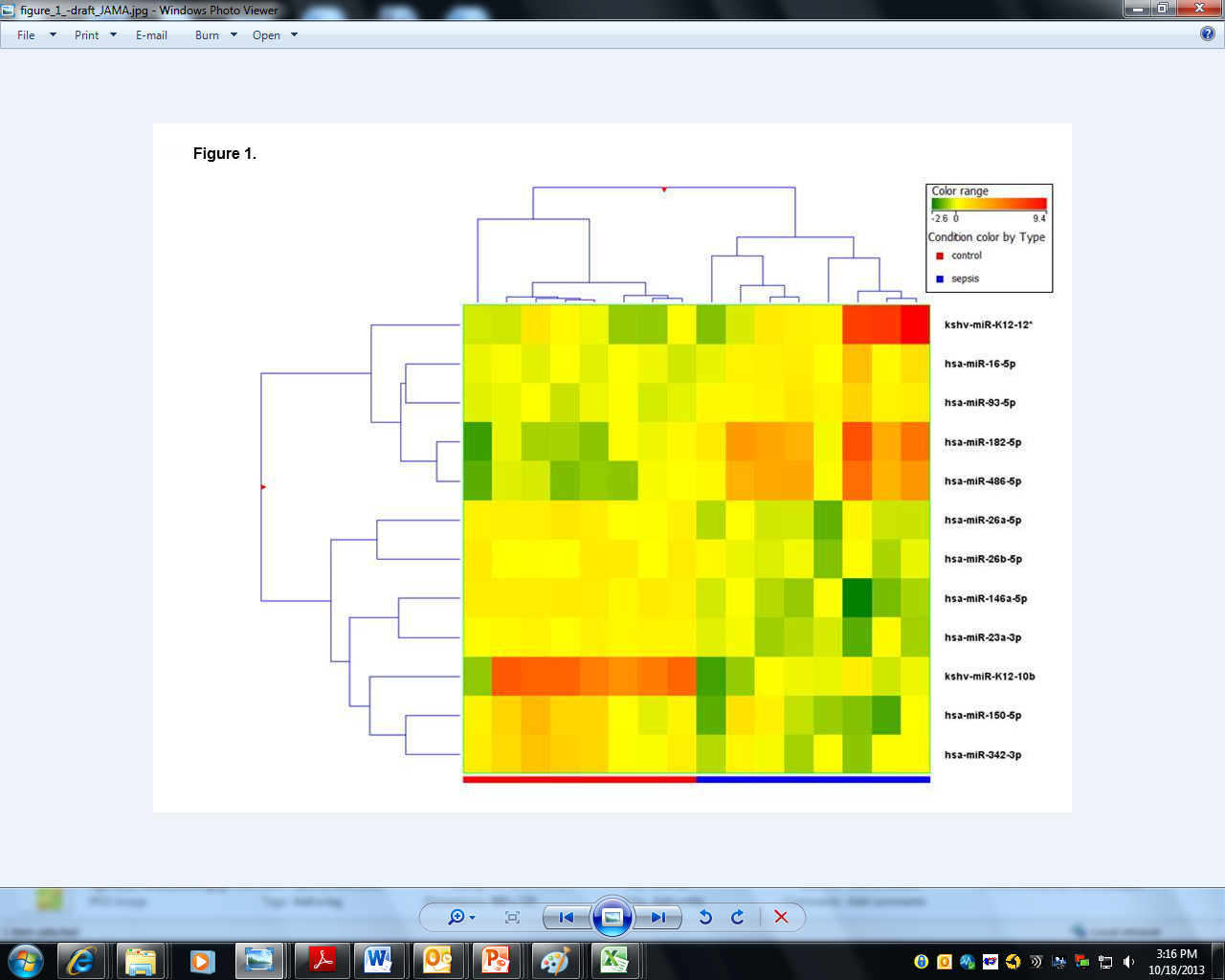


**Supplementary Figure 3.Cluster analysis of cellular and viral miRNAs expressed in leucocytes from sepsis patients versus normal healthy controls.** The clusters were generated based on microarray data using RNA from MNCs.


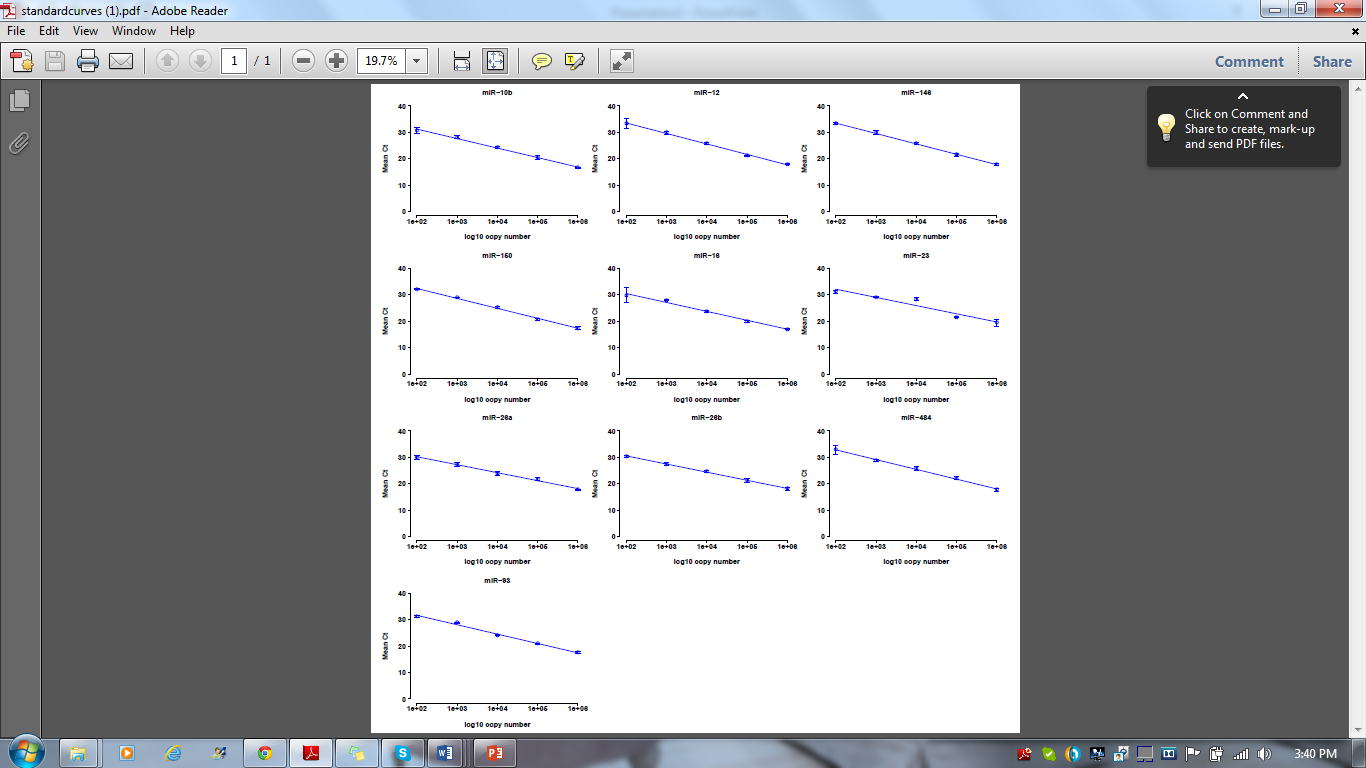


**Supplementary Figure 4.** Efficiency primer amplification for the selected microRNAs to adjust the background at the correct Ct.


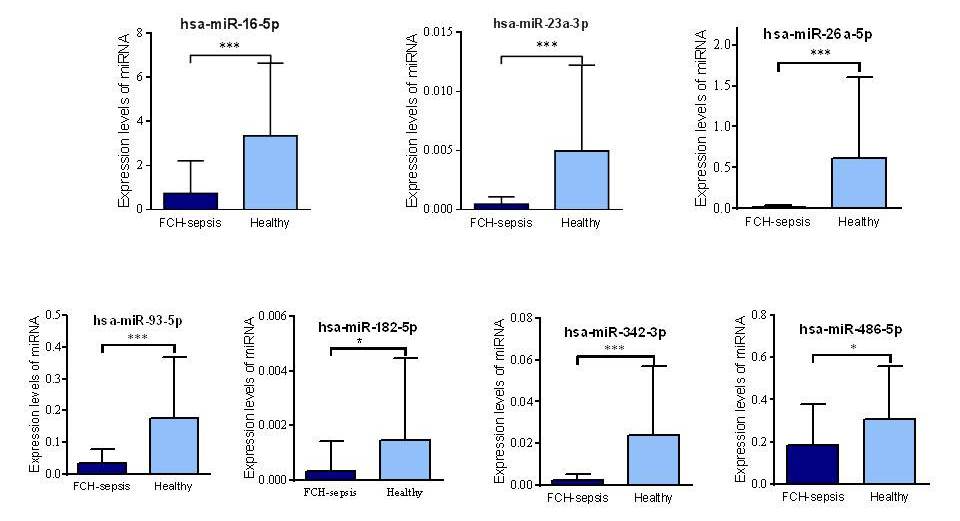


**Supplementary Figure 5. Plasma levels of cellular miRNAs detected by qRT-PCR.**Comparison of miRNAs between FCH sepsis patients (n=33) (Day 1) and healthy volunteers (n=53) (****P*<.0001, **P*<.05).


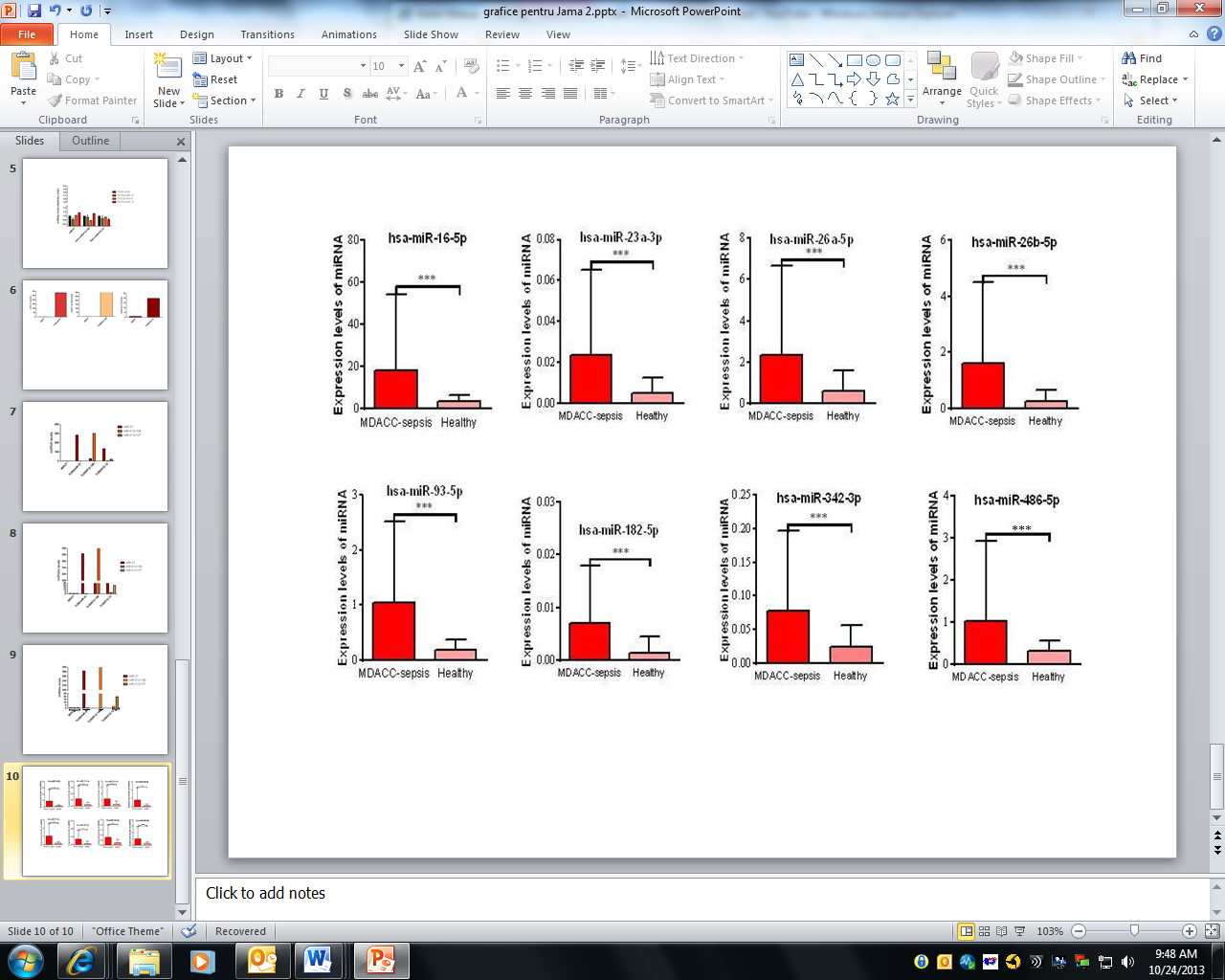


**Supplementary Figure 6. Plasma levels of cellular miRNAs detected by qRT-PCR.**Comparison of miRNAs expression between MD Anderson (MDACC) sepsis Patients (n=66) (Day1) and healthy Volunteers (n=53) (****P*<.0001).


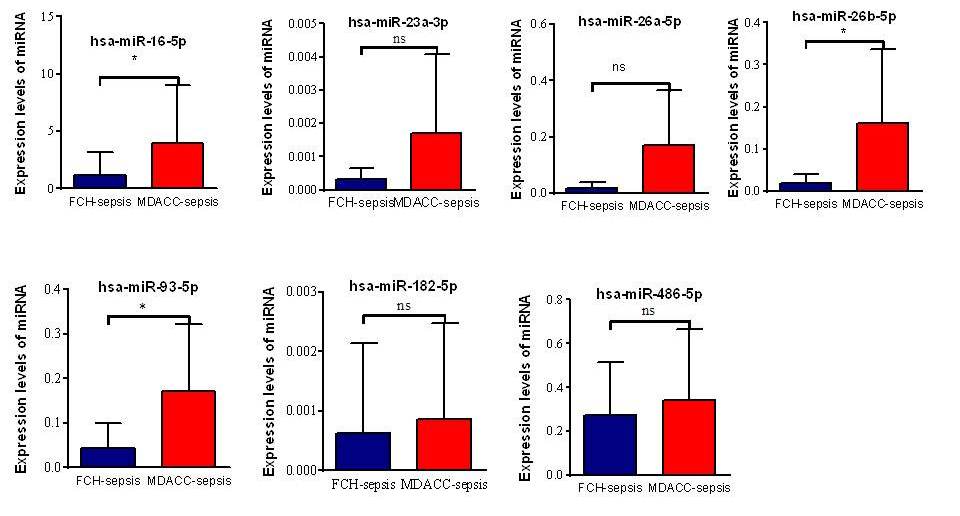


**Supplementary Figure 7. Comparison of miRNAs expression between FCH non-survivor sepsis patients and MD Anderson (MDACC) non-survivor sepsis patients.****P*<.05


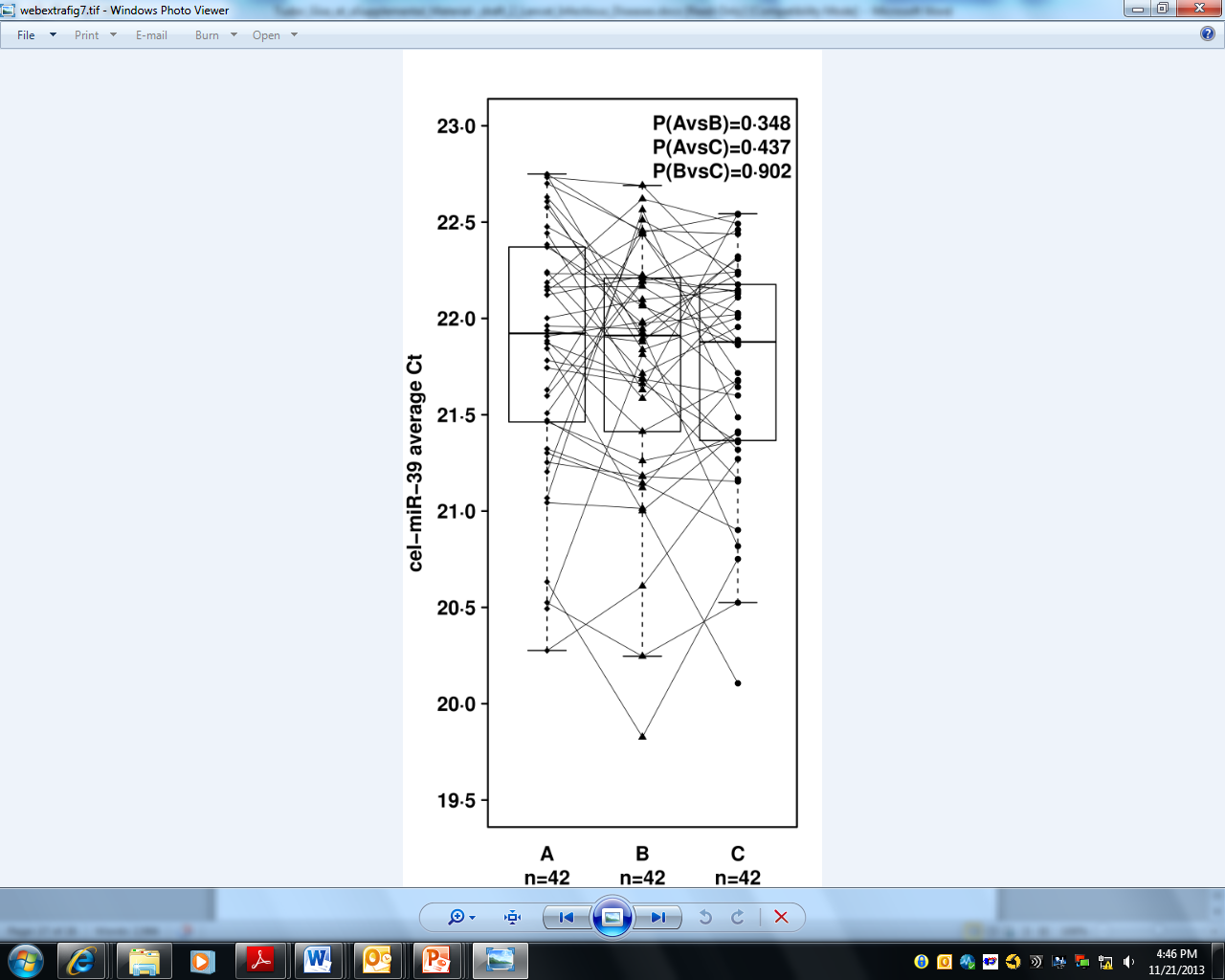


**Supplementary Figure 8.Ct values of cel-miR-39 for plasma samples in paired-wise comparison between preoperative phase, day 1 after surgery and day 7 after surgery.**

1. **b. c.**


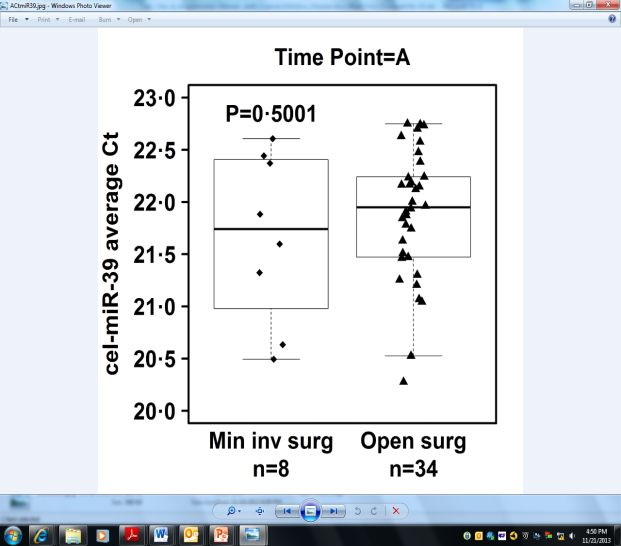

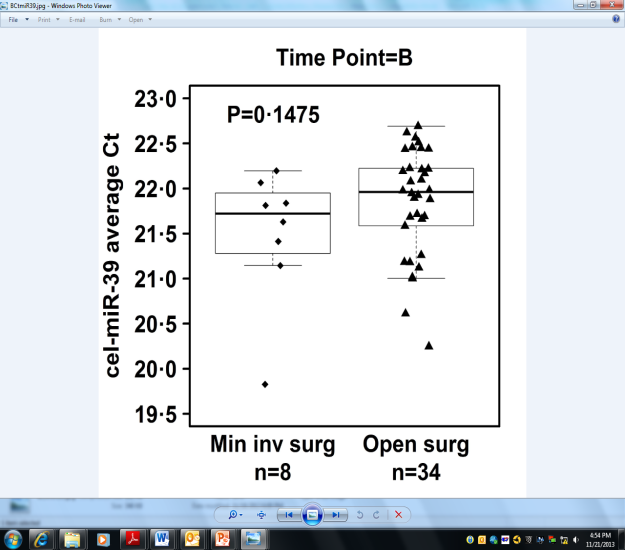

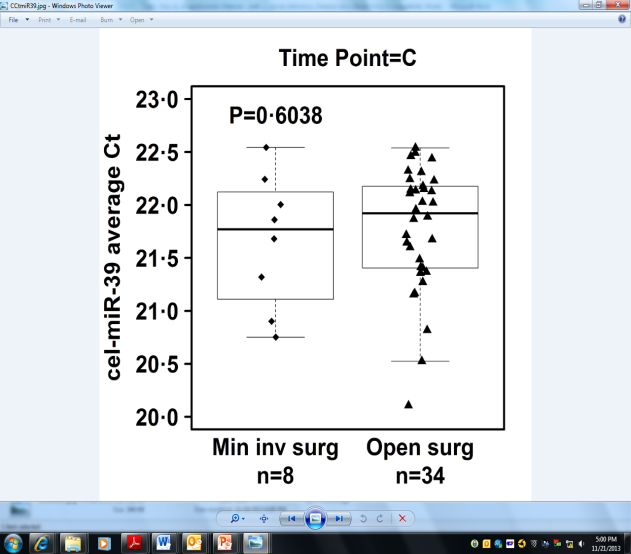


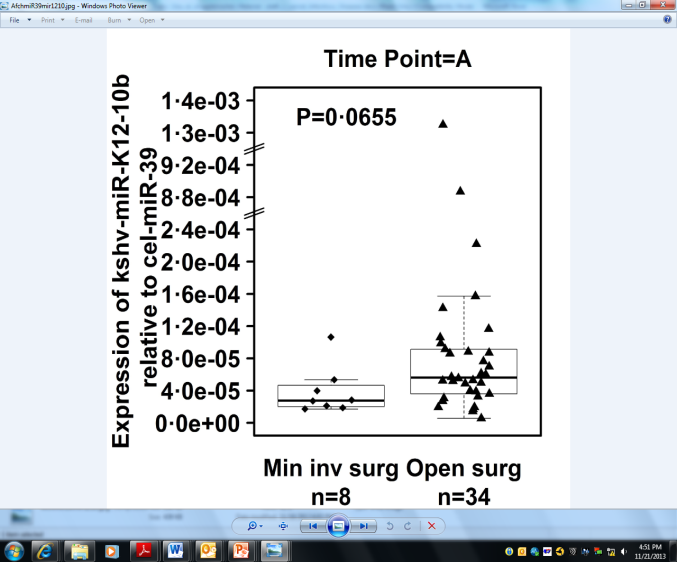

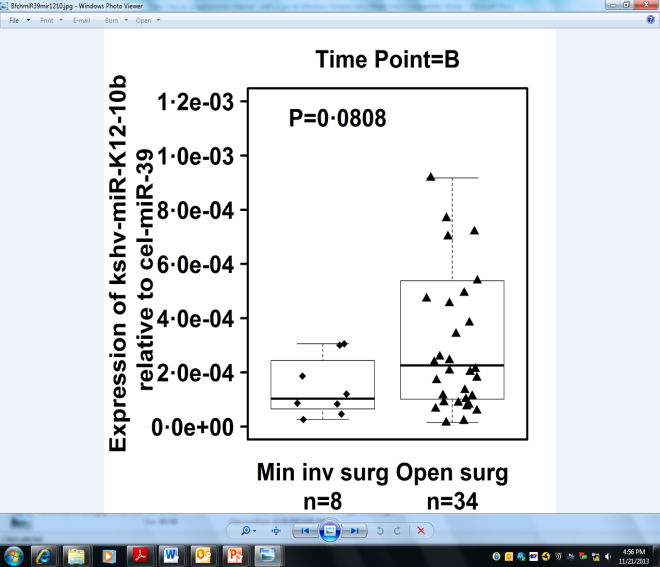

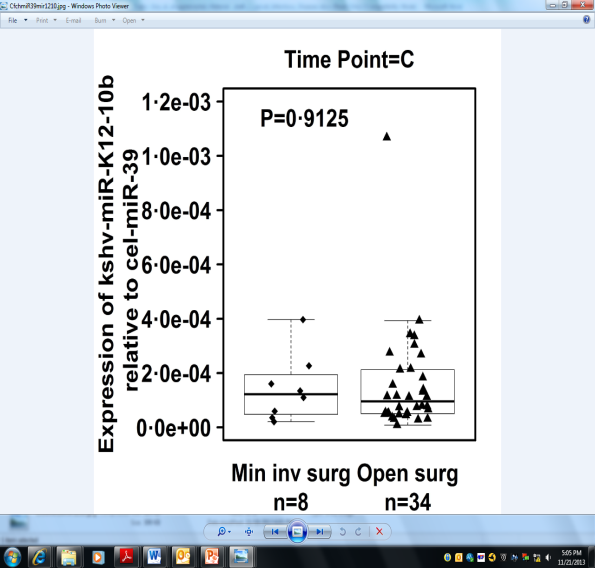


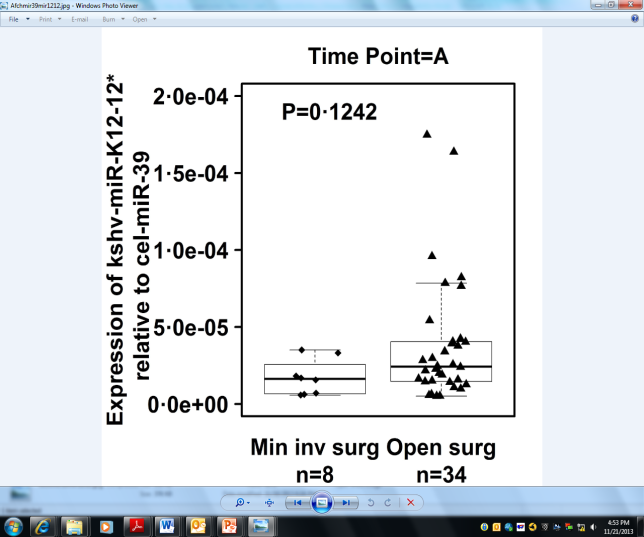

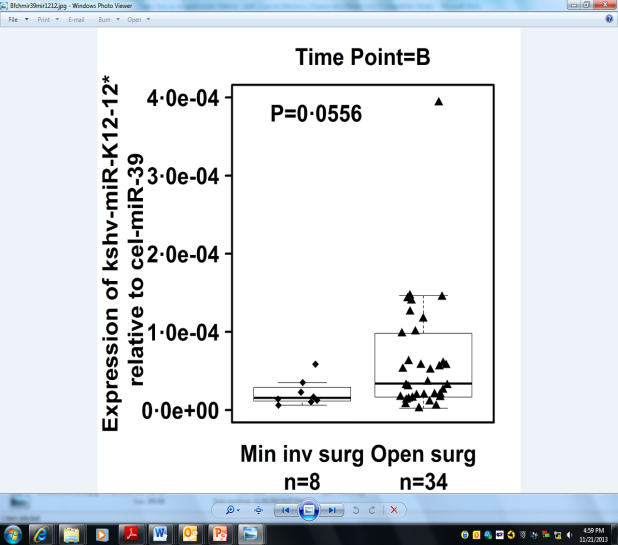

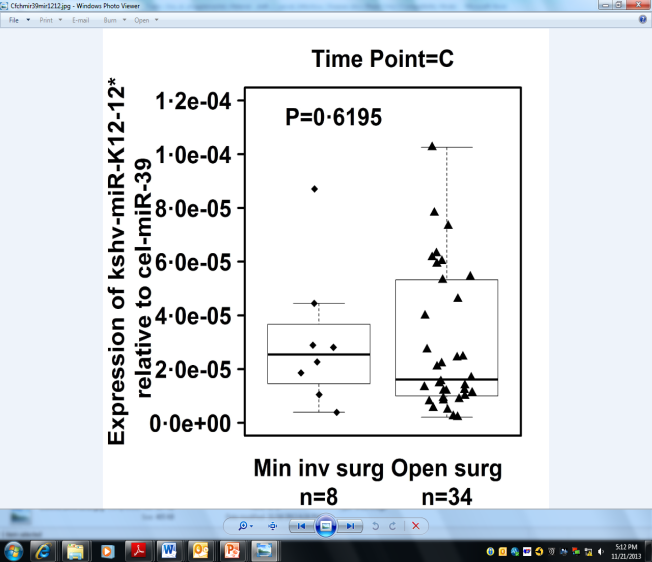


**Supplementary Figure 9. Differential expression of plasma KSHV miR-K12-10b and miR-K12-12* in patients who underwent open surgery and minimally invasive surgery.**(A) preoperative phase, (B) day 1 after surgery, (C) day 7 after surgery.

**a.**


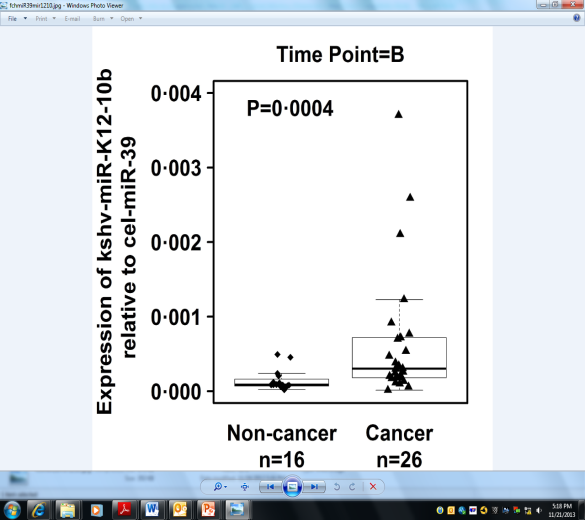

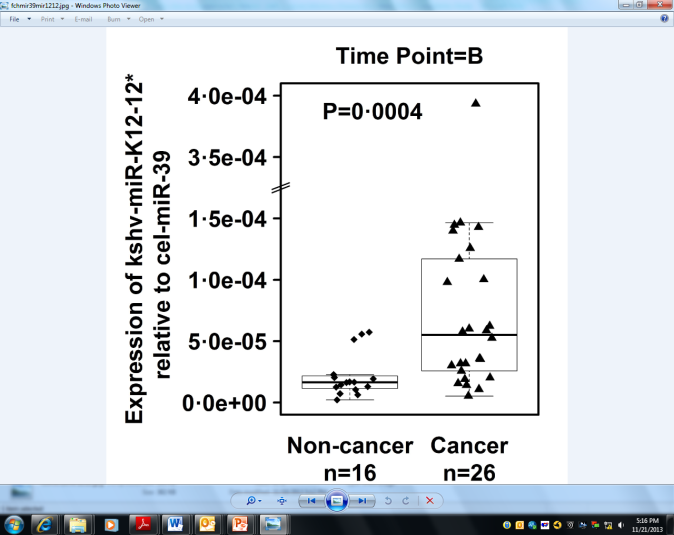

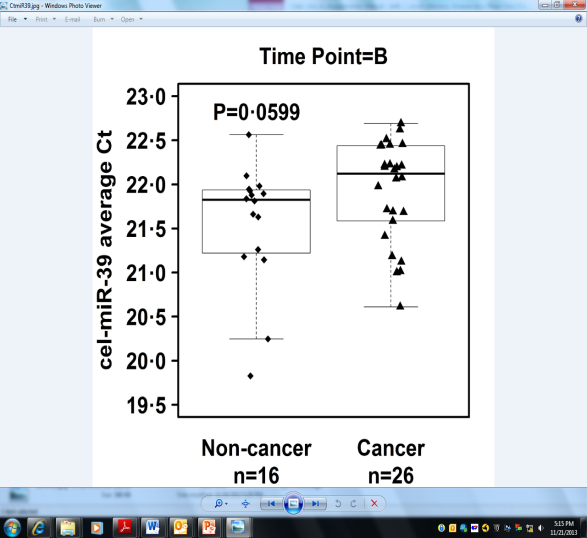


**b. c.**


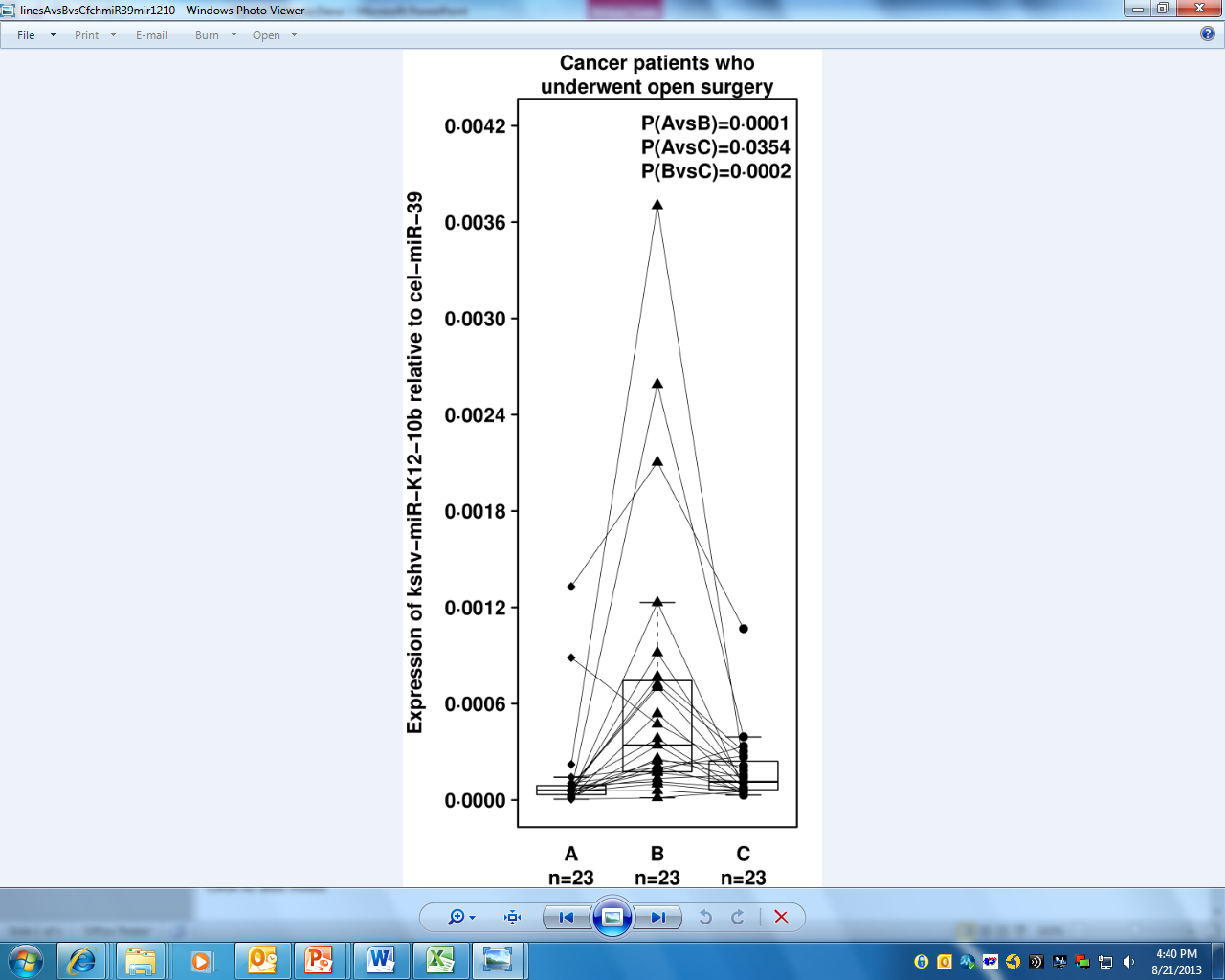

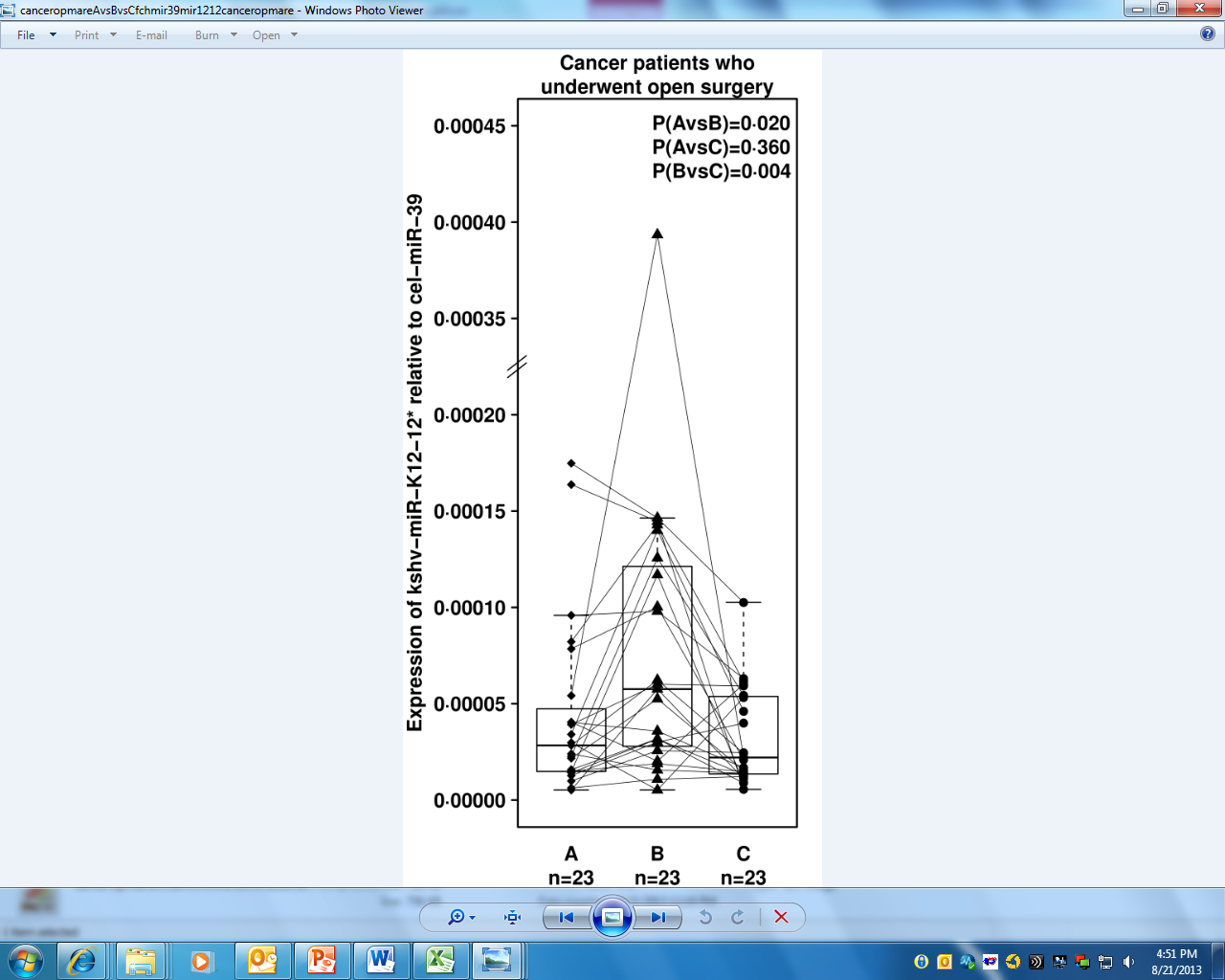

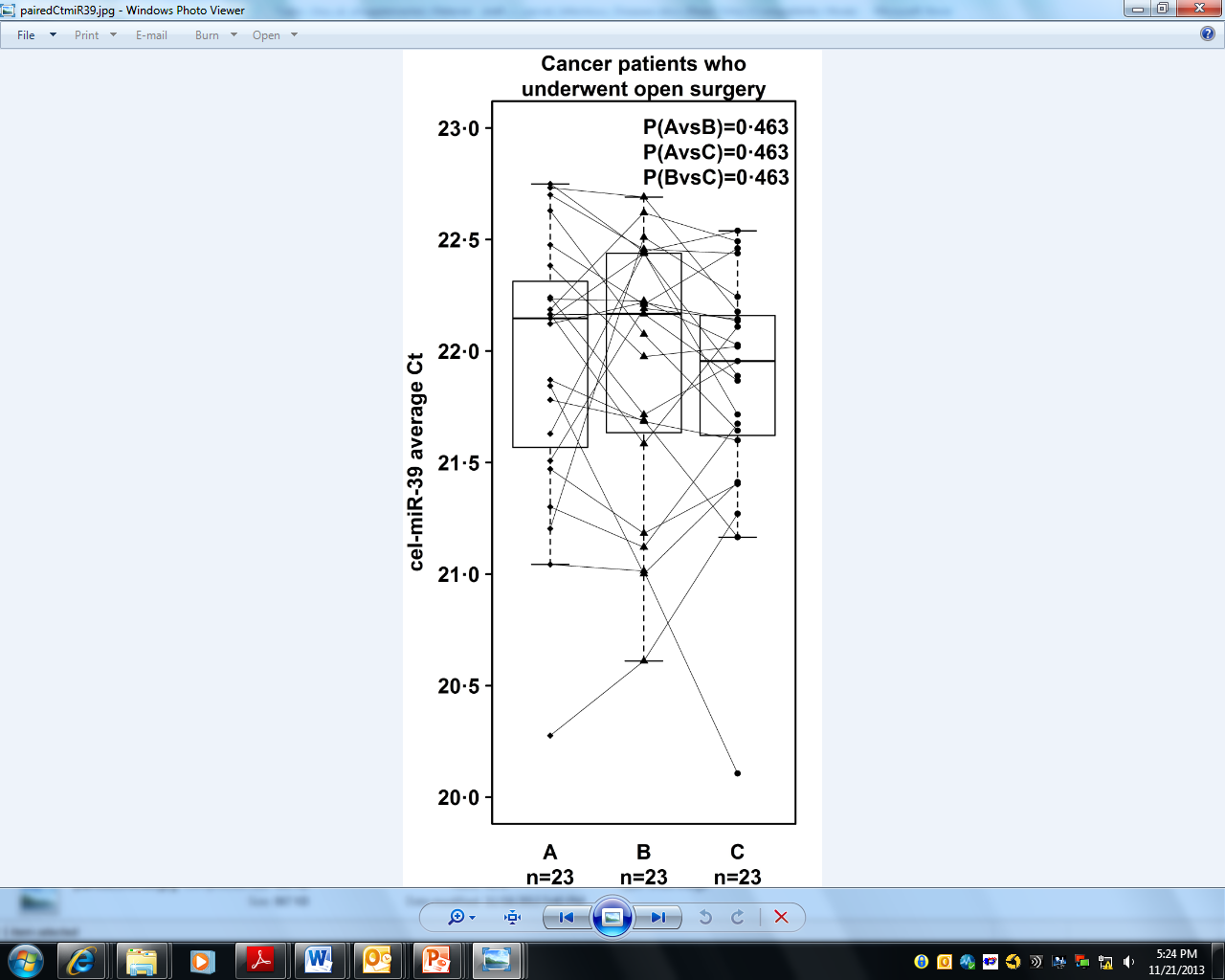


**Supplementary Figure 10.Differential expression of plasma KSHV-miR-K12-10b and miR-K12-12* in cancer and non-cancer patients.**(a) Differences in the expression of KSHV-miR-K12-10b on day 1 post surgery between cancer (n = 26) and non-cancer patients (n = 16). (b) Expression of KSHV-miR-K12-10b in cancer patients on day 1 post open surgery (n = 23) and the Ct values of cel-miR-39 for plasma samples in pair-wise comparison between preoperative phase, day 1 after surgery, and Day 7 after surgery.(c) The Ct values for cel-miR-39 on day 1 after surgery in cancer patients who underwent open surgery were similar. We used for this comparisons the relative expression of KSHV-miR-K12-10b and of KSHV-miR-K12-12* to cel-miR-39, which was stable across the studied groups.

**
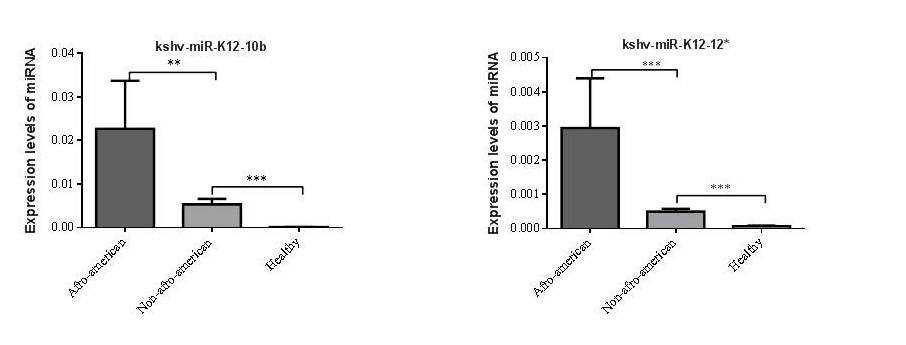
**

**Supplementary Figure 11. Differential expression of plasma miRNAs between Afro-Americans (n=11) and Non-Afro-Americans (n=88).**(****P*<.0001, ***P*<.001).

**
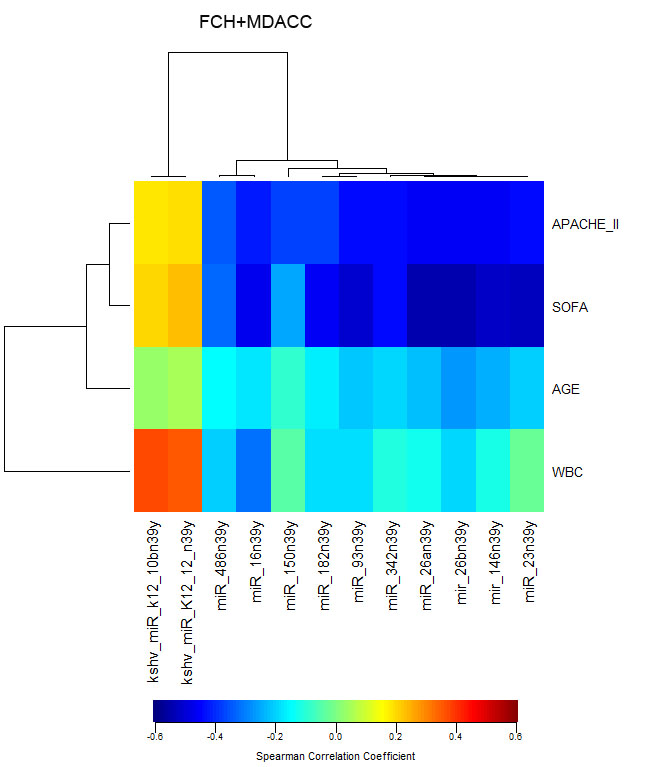
**

**Supplementary Figure 12.Correlations between patient demographic and clinical characteristics with miR expressions (test *P*-values and Spearman correlation coefficients).**

**
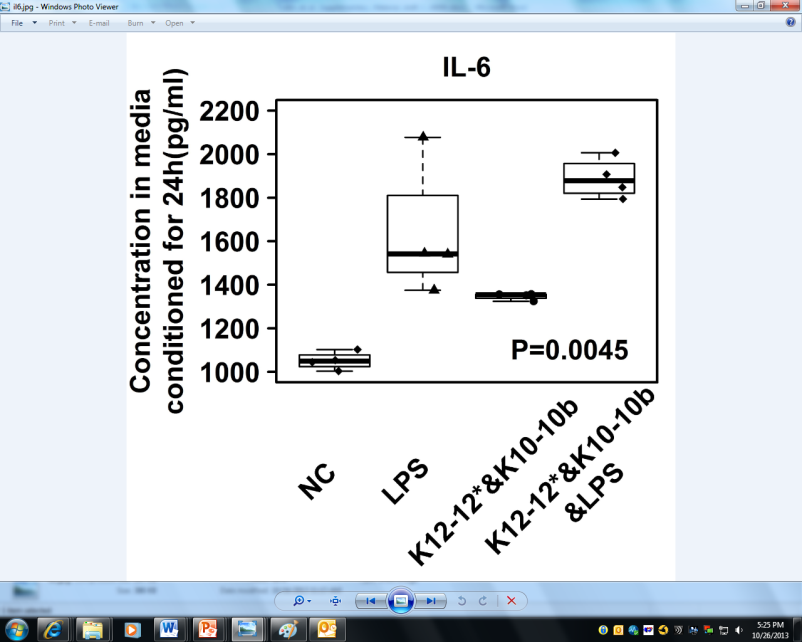
**

**Supplementary Figure 13.The IL-6 induction identified by a microbead-based immunosorbent assay platform.** Data was generated from U937 cells (data at 24 hours after Transfection). Ctrl = Negative Control;KSHV miRNAs = K12-12*+K12-10b;Both = K12-12*+K10b+LPS (K12-12* = KSHV-miR-K12-12*, K12-10b =KSHV- miR-K12-10b).

**
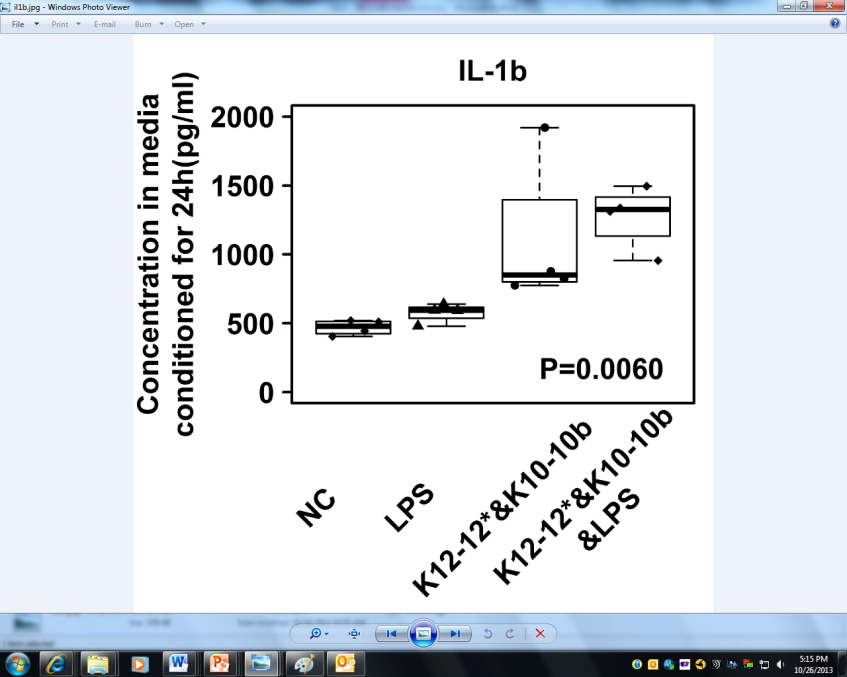
**

**Supplementary Figure 14.The IL-1b induction identified by a microbead-based immunosorbent assay platform.**Data were generated from U937 Cells data at 24 hours after Transfection). Ctrl = Negative Control;KSHV miRNAs = K12-12*+K12-10b;Both = K12-12*+K10b+LPS (K12-12* = KSHV-miR-K12-12*, K12-10b =KSHV- miR-K12-10b).

**a.**

**
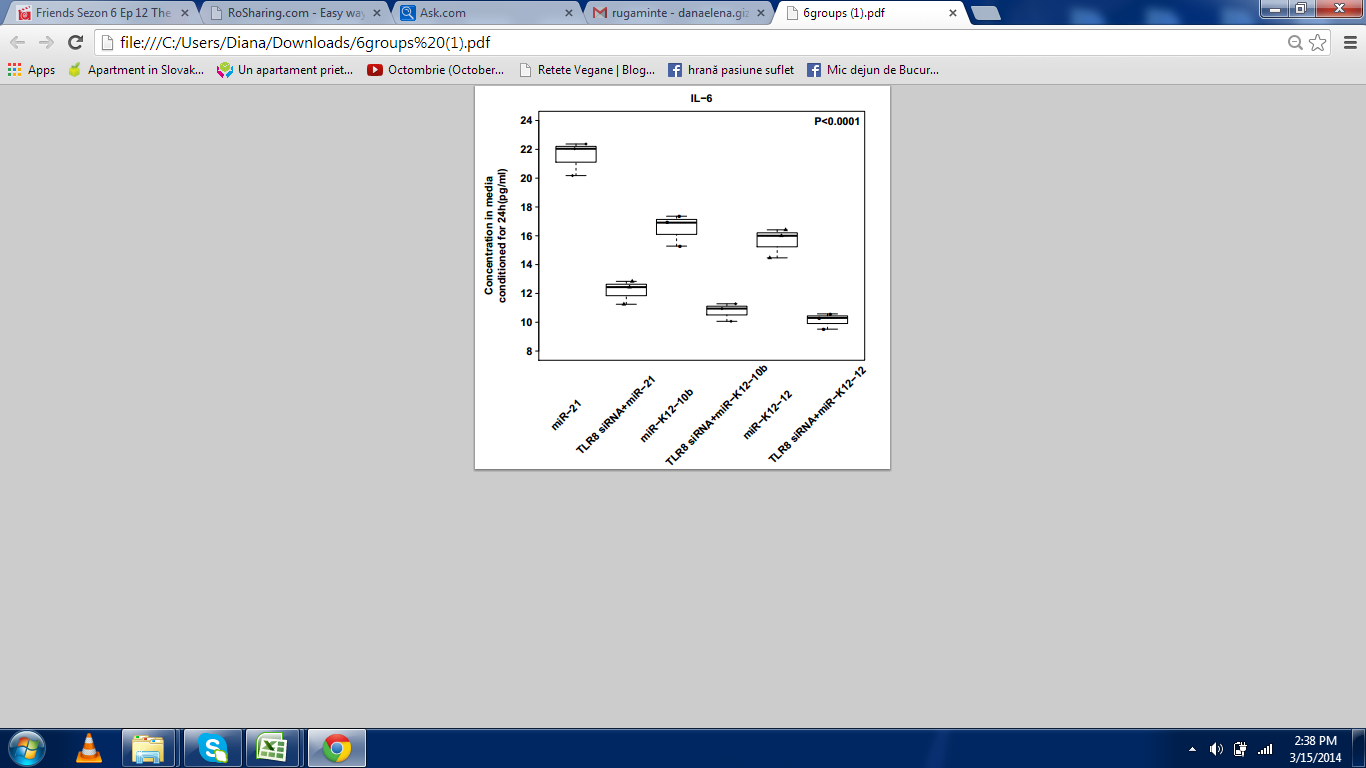
**

**b.**

**
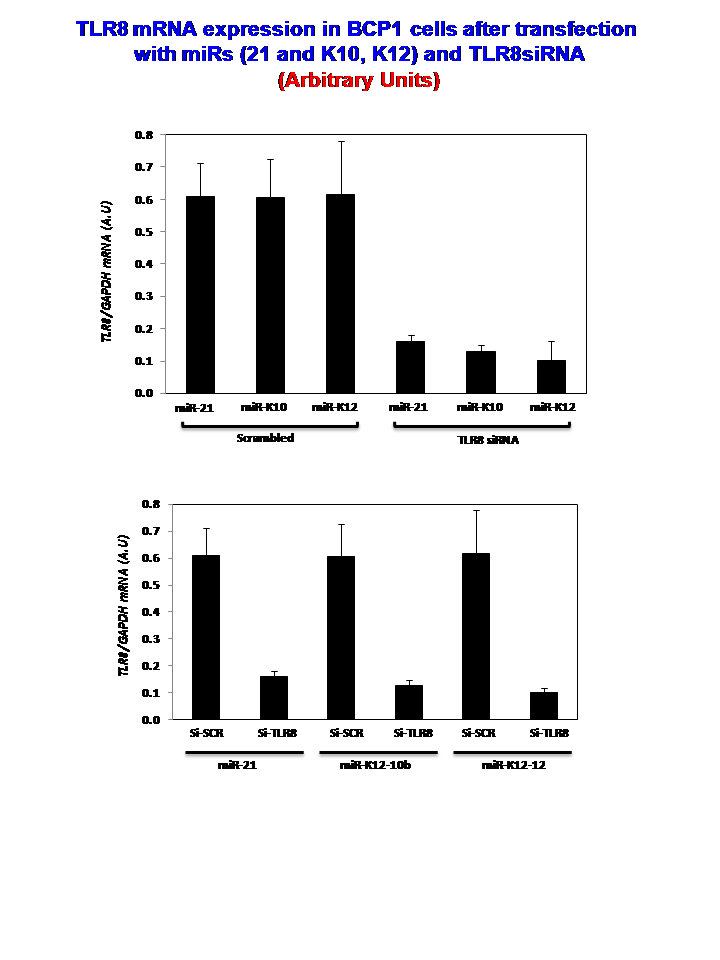
**

**Supplementary Figure 15. KSHV miRNAs are direct agonists of TLR 8 and they increase cytokine production when co-transfected in** BCP­1 clonal lymphoma cell line positive for KSHV. (a) KSHV-miR-K12-10b and KSHV-miR-K12-12* increase the secretion of IL-6 in BCP1 cells. b) TLR8 mRNA expression in BCP1 cells after transfection with miRs (21 and K-10b, K-12-12*) and TLR8 siRNA (miR-K12-12* = KSHV-miR-K12-12*, miR-K12-10b =KSHV- miR-K12-10b).

**Reference List:**

1. Vasilescu C, Rossi S, Shimizu M, Tudor S, Veronese A, Ferracin M, Nicoloso MS, Barbarotto E, Popa M, Stanciulea O, Fernandez MH, Tulbure D, Bueso-Ramos CE, Negrini M, Calin GA. MicroRNA fingerprints identify miR-150 as a plasma prognostic marker in patients with sepsis. PLoS. One. 2009; 4: e7405

2. Lopez DS, I, Galban S, Martindale JL et al. Identification and functional outcome of mRNAs associated with RNA-binding protein TIA-1. Mol Cell Biol. 2005;25(21):9520-9531
